# From Quinoline to Quinazoline‐Based *S. aureus* NorA Efflux Pump Inhibitors by Coupling a Focused Scaffold Hopping Approach and a Pharmacophore Search

**DOI:** 10.1002/cmdc.202100282

**Published:** 2021-06-26

**Authors:** Nicholas Cedraro, Rolando Cannalire, Andrea Astolfi, Gianmarco Mangiaterra, Tommaso Felicetti, Salvatore Vaiasicca, Giada Cernicchi, Serena Massari, Giuseppe Manfroni, Oriana Tabarrini, Violetta Cecchetti, Maria Letizia Barreca, Francesca Biavasco, Stefano Sabatini

**Affiliations:** ^1^ Department of Life and Environmental Sciences Università Politecnica delle Marche via Brecce Bianche 60131 Ancona Italy; ^2^ Current address: Department of Pharmacy University of Napoli “Federico II” via D. Montesano 49 80131 Napoli Italy; ^3^ Department of Pharmaceutical Sciences Università degli Studi di Perugia via del Liceo 1 06123 Perugia Italy

**Keywords:** medicinal chemistry, antibiotics, *Staphylococcus aureus*, NorA efflux pump inhibitors, quinazoline derivatives

## Abstract

Antibiotic resistance breakers, such as efflux pump inhibitors (EPIs), represent a powerful alternative to the development of new antimicrobials. Recently, by using previously described EPIs, we developed pharmacophore models able to identify inhibitors of NorA, the most studied efflux pump of *Staphylococcus aureus*. Herein we report the pharmacophore‐based virtual screening of a library of new potential NorA EPIs generated by an *in‐silico* scaffold hopping approach of the quinoline core. After chemical synthesis and biological evaluation of the best virtual hits, we found the quinazoline core as the best performing scaffold. Accordingly, we designed and synthesized a series of functionalized 2‐arylquinazolines, which were further evaluated as NorA EPIs. Four of them exhibited a strong synergism with ciprofloxacin and a good inhibition of ethidium bromide efflux on resistant *S. aureus* strains coupled with low cytotoxicity against human cell lines, thus highlighting a promising safety profile.

## Graphical abstract

**Resistance breakers**: By using scaffolds of approved drugs, we performed a scaffold hopping of the quinoline core coupled with a pharmacophore‐based virtual screen. The best hits were synthesized and evaluated as NorA efflux pump inhibitors (EPIs), and two 2‐arylquinazoline analogues were identified as potent NorA EPIs nontoxic toward human cells.

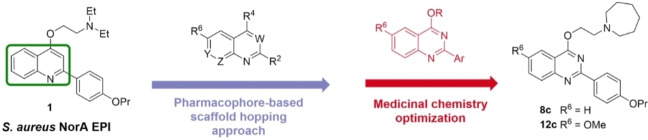

## Introduction

Antimicrobial resistance (AMR) represents a worldwide health issue prompting the World Health Organization (WHO) to declare it as one of the top 10 global health threats. Recent reports from WHO show that 10 million deaths will occur by 2050 if action is not undertaken.[^1^, ^2^, ^3^, ^4^, ^5^]

Microorganisms can acquire resistance by four main mechanisms: i) alteration of the target site, ii) enzymatic drug inactivation/modification, iii) decreased uptake or enhanced efflux of the drug and iv) biofilm formation.[^2^, ^6^, ^7^, ^8^, ^9^] Of note, the latter two constitute non‐specific defenses aimed at decreasing antibiotic concentration into microorganisms in turn allowing for the development of more specific mechanisms of resistance, thus representing the first microbial response upon antibiotic exposure. To this end, many approaches have been employed to fight the onset of AMR and among them, the discovery of antibiotic resistance breakers (ARBs) seems to be most promising over the development of novel antibacterials destined to generate new AMR. Indeed, the resistance often rises against compounds that have bactericidal or bacteriostatic effects and, for this reason, the strength of the ARBs lies on the lack of antibacterial activity combined with the ability to synergize with known antimicrobials.[^10^, ^11^, ^12^] Since overexpression of efflux pumps is one of the first mechanisms that microorganisms use to acquire resistance, the identification of efflux pump inhibitors (EPIs) represents a promising strategy to counteract AMR.

Based on their sequence similarity, microbial efflux pumps are classified into six different families: i) ATP‐Binding Cassette (ABC), ii) Major Facilitator Superfamily (MFS), iii) Multidrug And Toxic compound Extrusion (MATE), iv) Small Multidrug Resistance (SMR), v) Proteobacterial Antimicrobial Compound Efflux (PACE), vi) Resistance‐Nodulation‐cell Division (RND). In addition, depending on whether they use ATP hydrolysis (ABC) or a positive charged ion gradient to perform drug transport, they can be further divided in two classes.^[13]^ Of note, based on their expression and function, NorA (MFS) in *Staphylococcus aureus*,^[14]^ AcrAB‐TolC (RND) in *Escherichia coli*,^[15]^ MexAB‐OprM (RND) in *Pseudomonas aeruginosa*,^[15]^ and MacB (ABC) in *Escherichia coli* ^[16]^ are among the mostly involved in AMR.

*S. aureus*, a Gram‐positive bacterium responsible of a wide variety of human infections with clinical manifestation, has been included among the ESKAPE pathogens together with other superbugs (***E**nterococcus faecium, **S**taphylococcus aureus, **K**lebsiella pneumoniae, **A**cinetobacter baumannii, **P**seudomonas aeruginosa* and ***E**nterobacter species*). Indeed, *S. aureus* is known for its methicillin‐resistant strain (MRSA) generating high level of resistance and threatening the human health.[^9^, ^17^, ^18^, ^19^] The most studied efflux pump in *S. aureus* is the MFS NorA, which is responsible for the proton motive force (PMF)‐mediated extrusion of different antibacterials such as the fluoroquinolone ciprofloxacin (CPX).^[14]^ Recently, NorA has been linked to biofilm formation[^20^, ^21^] and to the development of more specific mechanisms of resistance through the increase of the mutation rate and the acquisition of plasmids.^[22]^

Due to difficulties in expressing and isolating the NorA protein and the consequent lack of 3D structures, the identification of NorA EPIs along the years relied on phenotypic screening of natural or synthetic compound libraries and drug repurposing,^[23]^ leading to different classes of NorA inhibitors, such as indole,[^24^, ^25^, ^26^] quinoline,^[27]^ boronic acid,[^28^, ^29^] chalcone,^[30]^ piperine^[31]^ and naphthyridine derivatives.^[32]^ However, none of them never reached clinical trials highlighting that further efforts are needed.

**Figure 1:**
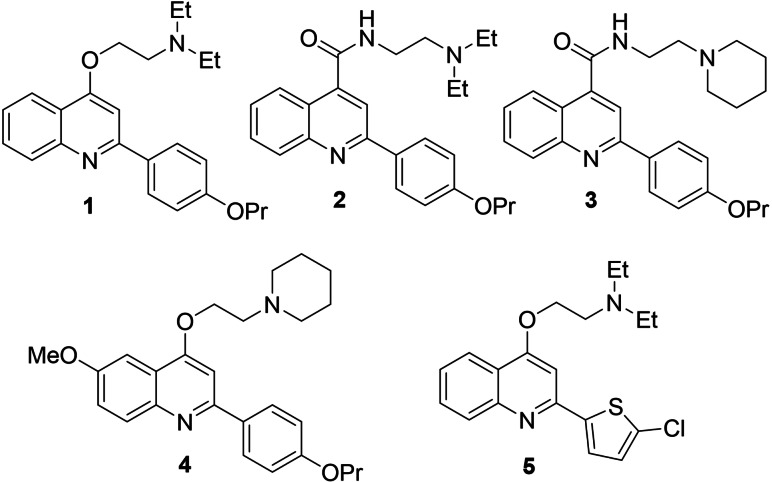
Chemical structures of previously reported quinoline NorA EPIs **1**–**5**.

In this context, we developed a class of quinolin‐4‐yloxy derivatives as NorA EPIs, exemplified by the representative compound **1** ^[33]^ (Figure 1), and more recently, we performed a scaffold hopping approach to enrich the array of NorA inhibitors.^[34]^ Briefly, building a library of 1456 small‐molecules (MW<300) obtained from the smart fragmentation of approved drugs^[34]^ and using compound **1** as the leading structure, we generated 6393 virtual hits which were analyzed in Phase^[35]^ by using, as queries, two pharmacophore models (ModB and ModC) previously developed to identify new NorA EPIs.^[36]^ Compounds showing a fitness score ≥1.7 for both models or ≥2.0 for at least one model were considered promising (167 derivatives). After visual inspection, scaffolds of four virtual hits were used to synthesize eight new compounds, based on four different cores, that led to the identification of quinoline‐4‐carboxamide analogues (**2** and **3** – Figure 1) with a potent NorA EPI activity resulting in the ability to synergize at low concentrations (1.56–3.13 μg/mL) with CPX against the resistant *S. aureus* strain SA‐1199B (*norA*+/GrlA mutation). From collected data, we also observed that a bicyclic scaffold, like the quinoline core of derivatives **1**–**3**, was essential to retain both NorA inhibition and synergism with CPX against SA‐1199B.

Herein, we report the identification of five virtual hits through a refinement of the previous scaffold hopping pharmacophore‐based screening approach focused on 6,6‐bicyclic cores. Starting from the five virtual hits, a set of 11 new molecules was designed, synthesized and biologically evaluated. After evaluation of NorA inhibition and synergism with CPX, the best series based on 2‐phenylquinazoline core was further investigated by functionalization of different positions of the core scaffold taking advantage from structure‐activity relationship (SAR) information available around the related quinoline class. Thus, additional alkylamino chains on the oxygen at C‐4 position and some moieties of previous reported NorA inhibitors (compounds **4** ^[37]^ and **5** ^[38]^ – Figure 1) were introduced on the new core affording nine new quinazoline derivatives.

## Results and Discussion

### Design

***In silico*** **selection of 6,6 bicyclic cores for scaffold hopping of quinoline based NorA EPIs**. We planned a scaffold hopping study in which the quinoline scaffold, represented by derivative **1**, was replaced by a library of different 6,6 bicyclic cores extracted from the DrugBank library.

**Figure 2:**
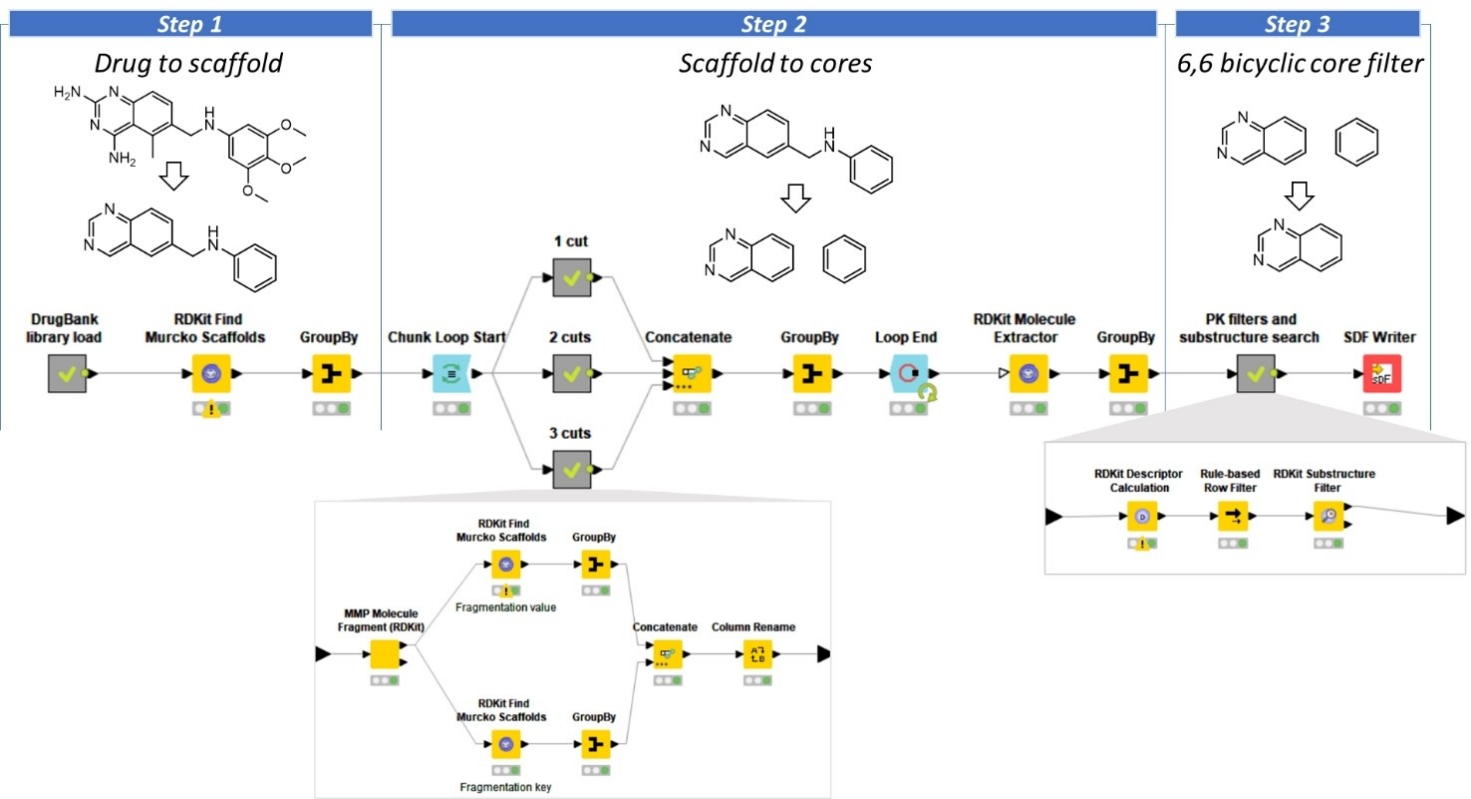
Overview of the developed KNIME workflow for the collection of 6,6 bicyclic cores (protocol details in the Experimental Section). In the figure is represented as example the fragmentation process for the approved drug Trimetrexate (DrugBank ID: DB01157).

A unique KNIME^[39]^ workflow was designed and built (Figure 2) to perform an exhaustive fragmentation of DrugBank library in order to obtain a unique collection of 6,6 bicyclic cores used in approved and experimental drugs (protocol details in the Experimental Section).

The workflow allowed the creation of a large database of drugs‐derived cores, from which 113 6,6 bicyclic systems were extracted and used as core library for our scaffold‐hopping strategy.

**Figure 3:**
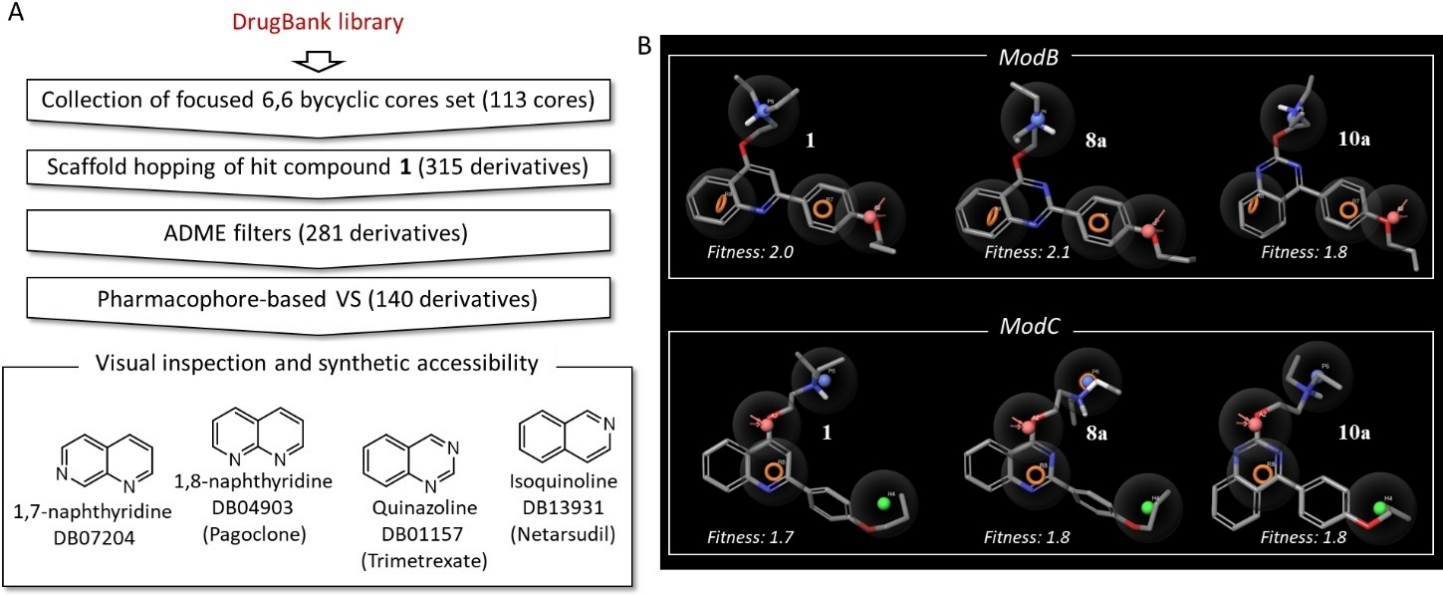
(A) Schematic overview of applied in‐silico workflow (B). Fitting of compounds **1**, **8 a** and **10 a** on ModB and ModC pharmacophoric models. Red sphere and vectors, H‐bond acceptor; blue sphere, positive charge; orange ring, aromatic ring; green sphere, hydrophobic moiety.

The scaffold hopping round generated 315 new compounds that were filtered based on absorption, distribution, metabolism and excretion (ADME) properties (see Experimental Section) and subsequently virtually screened in Phase^[35]^ by using the two pharmacophore models ModB and ModC as queries (Figure 3). Only compounds showing a fitness score ≥1.5 on both models were kept (140 molecules) and visually inspected, assessing both the ability of screened compounds in fitting the pharmacophore features and their synthetic feasibility.

The selected four cores, 1,7‐naphthyridine, 1,8‐naphthyridine, quinazoline and isoquinoline (Figure 3A), generated five virtual hits (**6 a–10 a** – Table 1) since the quinazoline core characterized two regioisomeric virtual hits (**8 a** and **10 a** – Figure 3B), having 2‐phenylquinazoline and 4‐phenylquinazoline scaffolds, respectively. In addition to the five identified virtual hits (**6 a–10 a**), a set of 5 close analogues having an ethylpiperidine chain in place of the ethyl‐*N*,*N*‐diethylamine was also designed (derivatives **6 b**–**10 b** – Table 1), taking advantage from the excellent results previously obtained with compounds **3** ^[34]^ and **4** ^[37]^ possessing this portion. However, during the synthesis of derivatives **7 a** and **7 b**, we only observed the formation of the respective *N*‐alkylated regioisomers **11 a** and **11 b** (structures and fitness scores on ModB and ModC in Table 1) which, when analyzed by Phase on ModB and ModC, did not reach the threshold of fitness values we imposed. The synergistic activity of **11 a** and **11 b** with CPX against SA‐1199B was investigated, but efforts were focused on the synthesis of the *O*‐alkylated derivative **7 b** in order to experimentally compare its synergistic activity with CPX with respect to the *N*‐alkylated analogue **11 b**. However, since no significant difference in terms of synergism with CPX against SA‐1199B was observed between **7 b** and **11 b**, synthesis of **7 a** was not carried out.

**Table 1:** Fitness scores on ModB and ModC, MIC values and CPX MIC fold reduction from synergistic assays at 12.5 μg/mL for compounds **6 a**, **6 b**, **7 a**, **7 b**, **8 a**–**f**, **9 a**, **9 b**, **10 a**, **10 b**, **11 a**, **11 b**, **12 a**–**c**, **13 a** and **13 b** and reference compound **1**.

Compd.		R^1^/R^4^	Fitness scores		SA‐1199		SA‐1199B	
			ModB	ModC	MIC [μg/mL]	Fold reduction^[a]^	MIC [μg/mL]	Fold reduction^[a]^
**6 a**	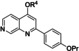	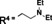	1.9	1.5	50	–^[b]^	50	4
**6 b**		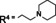	1.9	1.7	>50	–^[b]^	>50	4
**7 a**	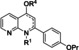	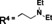	2.0	1.8	NT^*c*^	NT^[c]^	NT^[c]^	NT^[c]^
**7 b**		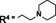	1.9	1.7	25	4	50	4
**11 a**		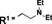	1.5	1.0	>50	4	>50	4
**11 b**		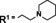	1.7	1.0	>50	–^[b]^	>50	4
**8 a**	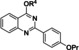	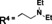	2.1	1.8	25	2	25	16
**8 b**		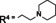	2.1	1.8	25	2	50	16
**8 c**		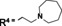	ND^[d]^	ND^[d]^	>50	–^[b]^	>50	16
**8 d**		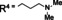	ND^[d]^	ND^[d]^	12.5	NT^[c]^	12.5	NT^[c]^
**8 e**		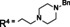	ND^[d]^	ND^[d]^	>50	–^[b]^	>50	4
**8 f**		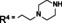	ND^[d]^	ND^[d]^	12.5	NT^[c]^	12.5	NT^[c]^
**9 a**	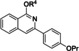	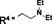	2.0	1.7	25	–^[b]^	25	2
**9 b**		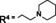	2.0	1.8	>50	4	50	2
**10 a**	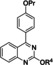	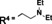	1.8	1.8	25	–^[b]^	25	8
**10 b**		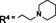	1.8	1.8	50	2	50	4
**12 a**	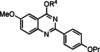	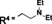	ND^[d]^	ND^[d]^	12.5	NT^[c]^	12.5	NT^[c]^
**12 b**		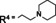	ND^[d]^	ND^[d]^	>50	–^[b]^	>50	8
**12 c**		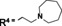	ND^[d]^	ND^[d]^	>50	–^[b]^	>50	8
**13 a**	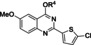	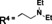	ND^[d]^	ND^[d]^	>50	2	>50	8
**13 b**		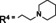	ND^[d]^	ND^[d]^	>50	2	>50	8
**1**			2.0	1.7	>50	–^[b]^	>50	4

[a] CPX MIC fold reduction from synergistic assays with compounds tested at the single concentration of 12.5 μg/mL; [b] No CPX MIC reduction. [c] NT=Not tested. [d] ND=Fitness scores not evaluated since the new designed analogues possessed the same scaffold as **8 a** and **8 b**.

**Design of further 2‐phenylquinazoline analogues**. As described in the following sections, evaluation of compounds **6 a**, **8 a**–**11 a** and **6 b**–**11 b** indicated the 2‐phenylquinazolines **8 a** and **8 b** as the best performing derivatives in terms of synergism with CPX against SA‐1199B, prompting us to further explore this series by functionalization of different positions of the core scaffold. Thus, taking advantage from SAR information available for parent quinoline class, additional alkylamino chains were introduced on the oxygen at C‐4 position (derivatives **8 c–f**) while, inspired by encouraging data for the previous hit compounds **4** and **5**, the methoxy group and the thiophene ring were considered for the C‐6 and the C‐2 positions, respectively, in the design of synthesized derivatives **12 a**–**c**, **13 a** and **13 b** (structures in Table 1).

### Chemistry

**Scheme 1:**
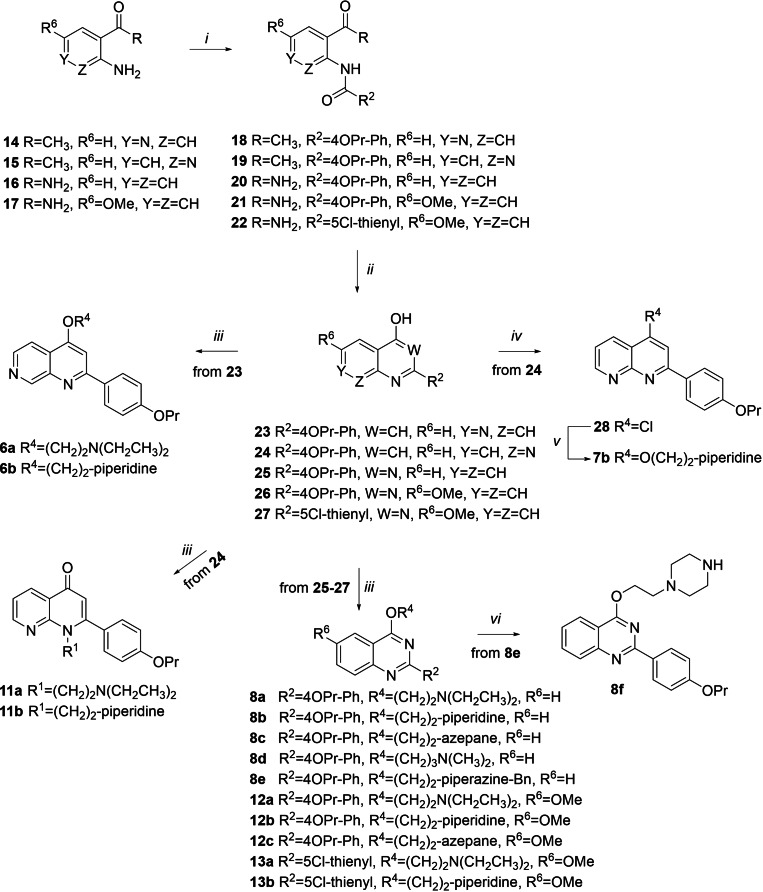
Reagents and condition: *i*) Et_3_N, dry THF, acyl chloride, rt – 60 °C, 3 h, 36–71 % or (for **18**) DMAP, dry dioxane, 4‐propoxy benzoyl chloride, 100 °C, MW, 10 min, 43 %; *ii*) NaOH, dry dioxane, 110 °C, MW, 10 min, 40 % or *t*BuOK, *t*BuOH, rt – 90 °C, 90 min – 3 h, 75–90 %; *iii*) K_2_CO_3_, chloroalkylamines, dry DMF, 80–90 °C, 1–5 h, 11–84 % or (for **8 c** and **8 d**) 90–100 °C, MW, 10–15 min, 14–84 %; *iv*) POCl_3_, 100 °C, 3 h, 80 %; *v*) 60 % NaH, 1‐(2‐hydroxyethyl)piperidine, dry DMF, rt, 3 h, 41 %; *vi*) 10 % Pd/C, ammonium formate, MeOH, reflux, 2 h, 14 %.

Compounds **6 a**, **6 b**, **7 b**, **8 a–f**, **11 a**, **11 b**, **12 a**–**c**, **13 a** and **13 b** were synthesized according to the synthetic procedure reported in Scheme 1. Amide coupling between acyl chlorides and variously substituted anilines **15**–**17** in dry THF, using Et_3_N as a base, afforded amide derivatives **19**–**22**. Similarly, 1‐(3‐aminopyridin‐4‐yl)ethanone **14** was reacted with 4‐propoxybenzoyl chloride but using DMAP as a base and exploiting microwave (MW) irradiation to give the benzamide derivative **18**. Then, by modifying a procedure reported by Brouwer *et al*.,^[40]^ **18** was cyclized in dry dioxane using NaOH as a base under MW irradiation into the naphthyridine derivative **23** in moderate yield.

In parallel, cyclization of **19**–**22** mediated by treatment with *t*BuOK in *t*BuOH gave the naphthyridine **24** and quinazolines **25**,^[41]^ **26** and **27**. Alkylation with 2‐chloro‐*N*,*N*‐dimethylethylamine hydrochloride or 1‐(2‐chloroethyl)piperidine hydrochloride of naphthyridine derivative **23** in dry DMF, and using K_2_CO_3_ as a base, under MW irradiation afforded the expected *O*‐alkylated derivatives **6 a** and **6 b**. On the other hand, alkylation of **24** under similar conditions led to *N*‐alkylation affording derivatives **11 a** and **11 b**. Synthesis of *O*‐alkylated derivative **7 b** entailed the chlorination of the naphthyridine derivative **24** to give **28** that was reacted with 1‐(2‐hydroxyethyl)piperidine in dry THF using NaH.

**Scheme 2:**
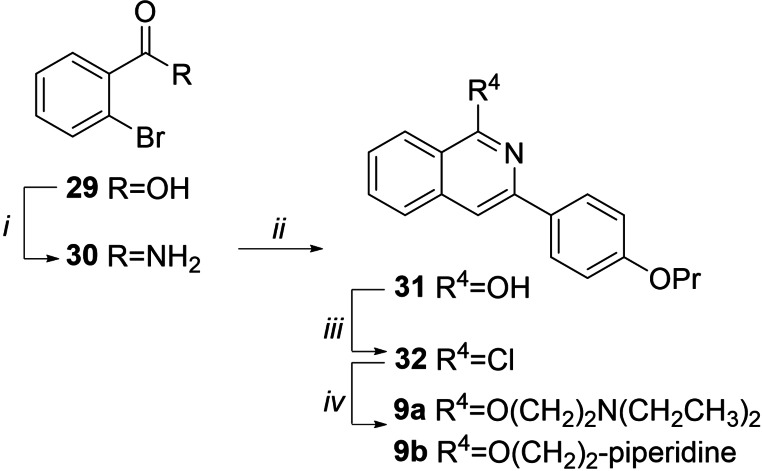
Reagents and condition: *i*) a) SOCl_2_, reflux, 1 h; b) aq. 33 % NH_3_, CH_3_CN, rt, 1 h, 95 %; *ii*) CuBr, 1‐(4‐propoxyphenyl)ethan‐1‐one, Cs_2_CO_3_, dry DMSO, 110 °C, 8 h, 30 %; *iii*) POCl_3_, reflux, 3 h; *iv*) 2‐(diethylamino)ethan‐1‐ol or 1‐piperidinethaanol, NaH, dry THF, reflux, 1–3 h, 50–53 %.

Alkylation of quinazoline scaffolds **25**–**27** with properly functionalized alkylamino chains in dry DMF, and using K_2_CO_3_ as a base, gave regioselective *O*‐alkylation affording derivatives **8 a**, **8 b**, **8 e**, **12 a**–**c**, **13 a** and **13 b**. Similarly, derivatives **8 c** and **8 d** were obtained by exploiting MW irradiation. Finally, benzyl removal of **8 e** in presence of ammonium formate and Pd/C as a catalyst afforded the piperazine derivative **8 f**. Compounds **9 a** and **9 b** were synthesized (Scheme 2) starting from the commercially available 2‐bromobenzoic acid **29** that was chlorinated in refluxing SOCl_2_ and then reacted with 33 % aq. NH_3_ in CH_3_CN to give amide **30** ^[42]^ in 95 % yield. By slightly modifying a procedure reported from Shi *et al*.,^[43]^ a copper(I)‐catalyzed α‐arylation followed by C−N bond formation between **30** and 1‐(4‐propoxyphenyl)ethan‐1‐one afforded the isoquinoline derivative **31**, in moderate yield. Chlorination in refluxing POCl_3_ gave **32** that immediately was reacted with 2‐(diethylamino)ethan‐1‐ol or 1‐piperidinethanol in presence of NaH and in dry THF to afford target compounds **9 a** and **9 b**.

**Scheme 3:**
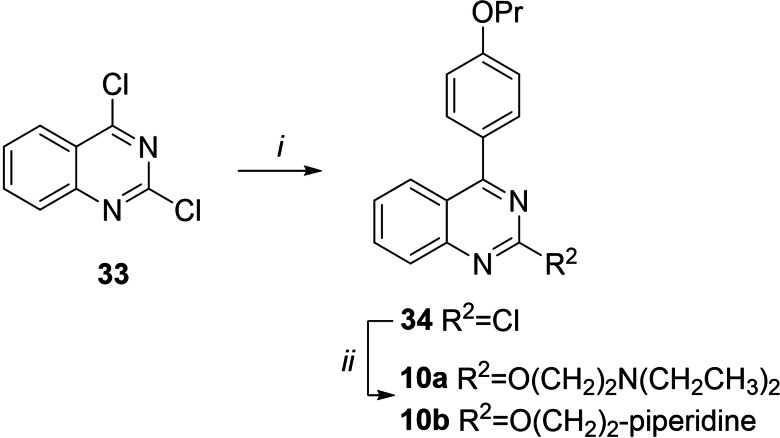
Reagents and condition: *i*) 4‐propoxyphenylboronic acid, Pd(PPh_3_)_4_, DME, aq. 2 M Na_2_CO_3_, MW, 100 °C, 15 min, 72 %; ii) 2‐(diethylamino)ethan‐1‐ol or 1‐piperidinethanol, NaH, dry THF, reflux, 1 h, 50–55 %.

Compounds **10 a** and **10 b** (Scheme 3) were synthesized starting from the commercially available 2,4‐dichloroquinazoline **33** by performing a Suzuki‐Miyaura cross coupling, using Pd(PPh_3_)_4_ as catalyst and aqueous 2 M Na_2_CO_3_ as base in DME exploiting MW irradiation, to regioselectively give derivative **34** in good yield. The structure of 4‐aryl regioisomer **34** was assigned by bidimensional ^1^H NMR experiments that highlighted NOE correlation between quinazoline H5 and 4’‐propoxyphenyl H2’/H6’. Reaction of **34** with 2‐(diethylamino)ethan‐1‐ol or 1‐piperidinethanol in dry THF and using NaH as base afforded target compounds **10 a** and **10 b**, respectively.

### Biological results

Initially, all compounds **6 a**, **6 b**, **7 b**, **8 a**–**f**, **9 a**, **9 b**, **10 a**, **10 b**, **11 a**, **11 b**, **12 a**–**c**, **13 a** and **13 b** were subjected to MIC evaluation on *S. aureus* strains SA‐1199 (wild‐type) and SA‐1199B (*norA*+/GrlA mutation). Since ideal EPIs should not possess antibacterial activity at low concentrations, derivatives **8 d**, **8 f** and **12 a** showing MIC values of 12.5 μg/mL were excluded from next synergistic assays, considering their moderate direct antibacterial effect. All other compounds exhibited MIC values ≥25 μg/mL against the two *S. aureus* strains. Of note, since SA‐1199 and SA‐1199B differ for the overexpression of NorA efflux pump and the presence of a mutation of DNA gyrase (target of fluoroquinolones), similar MIC values of tested compounds on both strains can suggest that our compounds are not NorA substrate neither DNA gyrase inhibitors.

Subsequently, compounds **6 a**, **6 b**, **7 b**, **8 a**–**c**, **8 e**, **9 a**, **9 b**, **10 a**, **10 b**, **11 a**, **11 b**, **12 b**, **12 c**, **13 a** and **13 b** were tested at the sub‐inhibitory concentration of 12.5 μg/mL in combination with scalar concentrations of CPX against SA‐1199 and SA‐1199B (Table 1). We included the 2‐phenylquinoline derivative **1** as a reference compound since it was used as quinoline template analogue to build pharmacophore models ModB and ModC. At 12.5 μg/mL, compound **1** showed a 4‐fold CPX MIC reduction against SA‐1199B while not exhibiting synergistic effect with CPX against SA‐1199.

Compounds able to reduce the CPX MIC at least 8‐fold against SA‐1199B and not more than 2‐fold against SA‐1199 were considered as promising NorA EPIs. Indeed, a compound that synergizes with CPX only on SA‐1199B while resulting ineffective on SA‐1199 likely exerts its synergistic activity through NorA inhibition. Therefore, compounds **8 a**–**c**, **10 a**, **12 b**, **12 c**, **13 a** and **13 b** matched these criteria resulting promising NorA EPIs; of note, compounds **8 a**–**c** showed the highest synergic effect exhibiting a 16‐fold CPX MIC reduction against SA‐1199B. In addition, the comparable MIC values on SA‐1199 and SA‐1199B suggested that the compounds are not substrate of NorA, thus the observed synergistic effect was not due to a substrate competition with the CPX during the NorA‐mediated efflux. Therefore, to facilitate the discussion, any synergistic effect with CPX against SA‐1199B will be considered as due to a NorA inhibition.

At this stage, having gained enough biological data around the new derivatives, a preliminary SAR can be drawn. As already observed in previous works, the presence of the alkylamino chain is essential but not sufficient to obtain NorA inhibition as well as the other three key features present in ModB and ModC (see Figure 3 for key features of both models). When quinoline core was replaced with 1,7‐ or 1,8‐naphthyridine systems, we observed a mild impact on the activity, yielding derivatives **6 a**, **6 b**, **7 b**, **11 a** and **11 b** that were able to reduce by 4‐fold the CPX MIC, likewise starting hit **1**.

Isoquinoline derivatives **9 a** and **9 b** did not exhibit any synergistic effect at 12.5 μg/mL with CPX against SA‐1199B, indicating that the preferred spatial arrangement is obtained when nitrogen atom is placed in position 1 with respect to the C‐4 *O*‐alkylamino chain, as in quinolin‐4‐yloxy **1**.

On the other hand, quinazoline analogues exhibited promising activities with the 4‐phenylquinazolines **10 a** and **10 b** exerting respectively 8‐ and 4‐fold CPX MIC reduction against SA‐1199B and the 2‐phenylquinazoline derivatives **8 a** and **8 b** showing the highest synergistic activity with CPX against SA‐1199B (16‐fold) and low or none effect against SA‐1199.

Focusing the discussion on the 2‐arylquinazolines, derivatives **8 c–f**, **12 a–c**, **13 a** and **13 b** showed different activity dependent from the substitution pattern. In a head a head comparison, the nature of the *O*‐alkylamino chains at C‐4 position impacted on intrinsic antibacterial activity or synergistic effect. Compounds **8 d** and **8 f**, owing to their intrinsic antibacterial effect at 12.5 μg/mL on both *S. aureus* strains, were excluded. Worth noting, compound **8 c** having an ethyl‐azepane moiety on the oxygen at quinazoline C‐4 position yielded a 16‐fold CPX MIC reduction, similarly to **8 a** and **8 b**. On the other hand, a benzyl‐piperazine‐ethyl moiety at the same position as in derivative **8 e** led to a decrease of the synergistic activity with CPX (4‐fold) but, interestingly, afforded MIC values on both SA‐1199 and SA‐1199B≥50 μg/mL, suggesting that a benzyl portion on the piperazine could mitigate the direct antibacterial effect observed for free piperazinyl derivative **8 f**.

Regarding C‐2 and C‐6 modifications, with the exception of **12 a** showing a direct antibacterial effect at 12.5 μg/mL on both the used *S. aureus* strains, the presence of a ‐OMe group at C‐6 position (derivatives **12 b** and **12 c**) as well as the combination of the introduction of a ‐OMe group at C‐6 and the replacement of the C‐2 *p*‐OPr‐phenyl moiety with a 5‐Cl thiophene portion (derivatives **13 a** and **13 b**) produced promising synergistic activities with an 8‐fold CPX MIC reduction against SA‐1199B. However, differently from C‐6‐OMe quinoline derivatives,^[37]^ we did not observe the expected improvement in NorA inhibition when a ‐OMe group was introduced at quinazoline C‐6 position.

Taken together, these results will guide us to better refine pharmacophore models, especially in order to narrow the requirements to identify potential NorA inhibitors.

**Figure 4:**
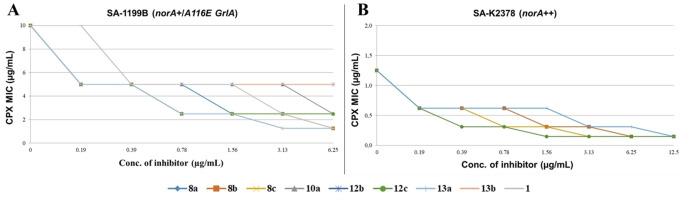
Checkerboard assays: **A)** CPX MIC reduction against SA‐1199B in the presence of increasing (from 0.19 to 6.25 μg/mL) concentrations of derivatives **8 a**–**c**, **10 a**, **12 b**, **12 c**, **13 a** and **13 b** and reference compound **1. B)** CPX MIC reduction against SA‐K2378 in the presence of increasing (from 0.19 to 12.5 μg/mL) concentrations of derivatives **8 b**, **8 c**, **12 c** and **13 a**. Assays performed in duplicate.

Motivated by these promising results, we selected all derivatives (**8 a**–**c**, **10 a**, **12 b**, **12 c**, **13 a** and **13 b**) that at 12.5 μg/mL showed at least a≥8‐fold CPX MIC reduction against SA‐1199B and low or no synergistic effect against SA‐1199 for more in‐depth checkerboard assays with CPX against SA‐1199B (Figure 4). Of note, derivatives **8 b**, **8 c**, **12 c** and **13 a** at as very low concentrations as 0.78 μg/mL produced an excellent synergistic activity reducing CPX MIC by 4‐fold reduction, while compound **8 a** needed a concentration of 1.56 μg/mL to retain a similar effect.

Unfortunately, compounds **10 a**, **12 b** and **13 b** did not show significant synergistic effect at concentration ≤6.25 μg/mL.

In addition, although less significant, all derivatives (with the exception of **13 b**) at 0.19 μg/mL reduced the CPX MIC against SA‐1199B by 2‐fold, thus showing that also at nanomolar concentrations these derivatives retain a certain degree of synergism with CPX. Since synergistic effect with CPX could occur by different ways, in particular by non‐specific mechanisms such as the permeability alteration or depolarization of the membrane of *S. aureus*, we further investigated the ability of our best compounds (**8 b**, **8 c**, **12 c** and **13 a**) in inhibiting NorA efflux by performing ethidium bromide (EtBr) efflux inhibition assays on SA‐1199B (Table 2) and checkerboard assays in combination with CPX (Figure 4) against SA‐K2378 overexpressing the *norA* gene.

**Table 2:** EtBr efflux inhibition (%) on SA‐1199B of compounds **8 b**, **8 c**, **12 c** and **13 a** at 50 μM. Evaluation of cell survival (%) of THP‐1 and A549 cell lines treated with **8 b**, **8 c**, **12 c** and **13 a** at 0.78 and 6.25 μg/mL.

Compd.	EtBr efflux inhib. at 50 μM (±SD)^[a]^	Cell survival (%)(±SD)^[b]^		
		Compd. Concentration [μg/mL]	THP‐1 cells	A549 cells
**8 b**	78.4±10.2	0.78	90.6±27.8	116.8±7.7
		6.25	90.1±5.3	136.3±8.1
**8 c**	79.4±16.1	0.78	92.5±11.5	124.8±7.3
		6.25	89.8±9.9	123.7±7.4
**12 c**	66.7±7.8	0.78	101.4±12.9	116.8±6.11
		6.25	84.9±9.4	116.2±4.7
**13 a**	68.2±13.2	0.78	93.8±19.0	109.1±4.0
		6.25	84.1±19.0	98.7±5.9

[a] SD, standard deviation; assays were conducted in triplicate performing two technical replicates for each biological replicate evaluated; [b] SD, assays were performed in biological and technical duplicate.

EtBr is a known substrate of NorA, commonly used to evaluate NorA inhibition due to its ability to intercalate bacterial DNA and resulting fluorescent only when inside the bacterial cells.^[44]^ As a result, fluorescent *S. aureus* cells will indicate a high concentration of EtBr inside the cells indirectly proving a strong NorA inhibition. Therefore, after loading SA‐1199B with EtBr, we treated them with 50 μM of each of our compounds and evaluated fluorescent changes for 5 minutes, thus calculating a percentage of EtBr efflux inhibition. Interestingly, all tested compounds (**8 b**, **8 c**, **12 c** and **13 a**) highlighted an EtBr efflux inhibition ≥65 % (Table 2), thus disclosing a strong interference with the process of NorA efflux.

Since the EtBr efflux assay represents a phenotypic screening performed in only 5 minutes, a further confirmation of NorA inhibition by our compounds was given by i) checkerboard assays in combination with CPX on SA‐K2378 (*norA*+) and ii) synergistic assays combining 12.5 μg/mL of each compound with scalar concentrations of CPX on SA‐K1902 (*norA−)*. To clarify, both strains display a basal expression of all efflux pumps present in *S. aureus* strains and therefore only differ for the absence (SA‐K1902) or the overexpression (SA‐K2378) of NorA. Accordingly, those compounds able to restore the CPX MIC on SA‐K2378 at the same level of the CPX MIC when tested alone on SA‐K1902 likely exert a specific inhibition of NorA efflux. Indeed, any nonspecific effect such as bacterial membrane alteration or depolarization would result in a synergistic effect with CPX also against SA‐K1902. Firstly, we evaluated MIC values of compounds **8 b**, **8 c**, **12 c** and **13 a** on SA‐K1902 and SA‐K2378 and observed that, with the exception of **13 a** (MIC=12.5 μg/mL), all compounds showed MIC values higher than 50 μg/mL. Subsequently, we performed checkerboard assays on SA‐K2378 for all four compounds and observed that at 1.56 μg/mL, they were able to reduce the CPX MIC by 4‐fold thus restoring the CPX MIC at the same level of the MIC of the CPX alone against SA‐K1902. In addition, **8 c**, **12 c** and **13 a** retained the same degree of synergism also at 0.78 μg/mL. In parallel, synergistic assays of **8 b**, **8 c** and **12 c** at 12.5 μg/mL and **13 a** at 6.25 and 3.13 μg/mL (reduced concentrations were used because **13 a** showed a MIC value of 12.5 μg/mL) on SA‐K1902 in combination with CPX highlighted the lack of any synergistic effect. Therefore, these data confirmed that the synergistic activity of all four compounds was probably due to a specific inhibition of NorA.

To further investigate the biological profile of compounds **8 b**, **8 c**, **12 c** and **13 a**, cytotoxicity evaluation on THP‐1 and A549 cell lines was carried out at different concentrations (0.78 and 6.25 μg/mL – Table 2). All tested compounds did not reduce A549 cell survival at all and showed only a negligible effect on THP‐1 cells. Worth noting, at 0.78 μg/mL, a concentration at which all four compounds show a remarkable synergistic effect with CPX (4‐fold CPX MIC reduction) against SA‐1199B, none of compounds caused a cell survival reduction higher than 10 % on THP‐1 cells.

In addition, THP‐1 cells retained 84 % survival also at the highest compound concentration tested of 6.25 μg/mL, which is 8‐fold greater than active concentration, thus showing a potential for future animal studies aimed at proving the efficacy of NorA EPI in *in vivo* models. These results clearly highlight that the replacement of 2‐phenylquinoline scaffold with 2‐arylquinazoline led to compounds with promising *in vitro* safety profiles.

**Figure 5:**
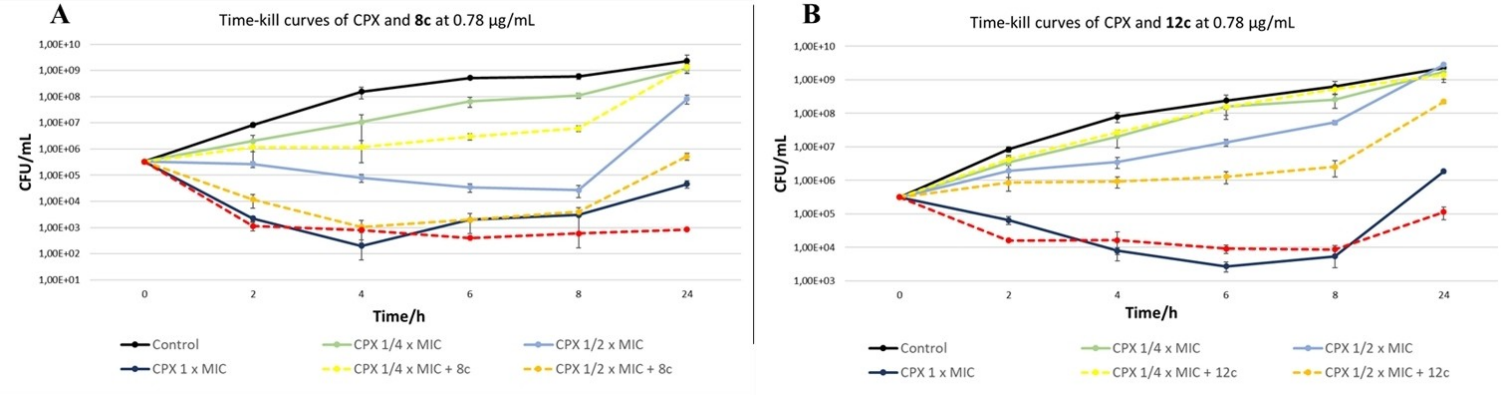
Time‐kill curves of CPX at 1/4 x, 1/2 x and 1 x MIC alone or in combination with compound **8 c** (A) and **12 c** (B) at 0.78 μg/mL against *S. aureus* strain SA‐1199B. Assays were performed testing at each time points 4 dilutions in duplicate.

Given the promising synergism with CPX in checkerboard assays, the activity of compounds **8 c** and **12 c** was further evaluated in time‐kill curve assays against *S. aureus* SA‐1199B in association with CPX, thus monitoring bacterial growth within 24 hours. Although tested at the low concentration of 0.78 μg/mL, when in combination with CPX at 1/4 ×MIC, compound **8 c** was able to enhance CPX activity (1–1.5 log decrease of survivors compared to the antibiotic alone) from 4 to 8 hours of treatment. The combination compound/CPX at 1/2 ×MIC exerted a bactericidal effect comparable to that of CPX alone at 1×MIC, while the combination compound/CPX at 1×MIC was able to further potentiate the activity at 24 hours of treatment, compared to antibiotic alone (Figure 5). When tested in combination with compound **12 c** at 0.78 μg/mL, CPX bactericidal activity over time seems to be potentiated mostly at a concentration corresponding to 1/2 ×MIC (about 1 log decrease of CFU/mL from 6 hours of treatment). In combination with CPX 1×MIC, compound **12 c** was able to enhance bactericidal activity at 24 hours of treatment, while no difference between presence and absence of compound was observed at a CPX concentration 1/2 ×MIC (Figure 5). Taken together, these results show the advantages of a combination between CPX and a NorA EPI, highlighting as, in presence of non‐antibiotic compounds **8 c** or **12 c**, the use of a reduced CPX concentration can reach the same bactericidal effect on the resistant *S. aureus* SA‐1199B strain within 24 hours.

## Conclusion

In this work, we reported the bicyclic scaffold hopping strategy of the quinoline core, present in previously identified *S. aureus* NorA EPIs. Using an *in silico* aided scaffold hopping combined with a pharmacophore virtual screening, we designed, synthesized and tested as NorA EPIs a series of 19 new derivatives having five different 6,6‐ring systems. Four novel 2‐arylquinazoline derivatives (**8 b**, **8 c**, **12 c** and **13 a**) turned out to strongly synergize with CPX at a concentration as low as 0.78 μg/mL against the resistant SA‐1199B (*norA+*), without any effect on wild type SA1199. Synergistic effect was indirectly related to NorA inhibition by performing EtBr efflux inhibition assays on SA‐1199B and checkerboard assays against *S. aureus* strains differing for the expression/deletion of the *norA* gene (SA‐K2378 and SA‐K1902). In addition, all four compounds did not exhibit significant human cell toxicity, showing a potential for future animal studies aimed at proving the efficacy of NorA EPI in *in vivo* models. Finally, the two best compounds **8 c** and **12 c** exhibited a high potential in synergizing with CPX against SA‐1199B also when evaluated by time‐kill curve analyses.

Besides the identification of new potent NorA EPIs, the aim of this study entailed the refinement of the SAR around the quinoline core, a scaffold widely explored by us in the past in order to identify NorA EPIs. Actually, quinoline core is present in derivative **1** that we previously used as quinoline representative compound to build NorA EPIs pharmacophore models. Data obtained in this work from the replacement of the quinoline with five different scaffolds will improve these models. Indeed, we observed that for retaining NorA inhibition activity: *i*) an extended aromatic portion in the central scaffold is needed^[34]^ with quinoline and quinazoline scaffolds preferred over 1,7‐naphthyridine and 1,8‐naphthyridine; *ii*) nitrogen at 1‐position of the quinoline core is essential as demonstrated by the lack of activity of isoquinoline derivatives **9 a** and **9 b**; iii) nitrogen at 3‐position (quinazoline analogues) can improve NorA EPI activity and in parallel reduce human cell toxicity. Therefore, these new findings will be used to build new updated pharmacophoric models that will be useful to perform drug repositioning studies and virtual screening campaigns to identify new NorA EPIs and also, as recently demonstrated,^[45]^ novel nontuberculous mycobacteria efflux inhibitors.

## Experimental Section

### In silico scaffold hopping

The DrugBank library (version 5.1.7) was downloaded and submitted to LigPrep.^[46]^ The neutral form of the ligands was prepared, and the tautomeric states was generated using Epik.[^47^, ^48^] Furthermore, at most 32 stereoisomers per ligand and three lowest energy conformations per ligand ring were produced. Where not defined, all the chiral form of each stereocenter was produced. Then, the prepared library was submitted to an in‐house developed KNIME workflow to extract for each molecule the corresponding scaffold. In the same workflow, each scaffold was fragmented to further extract each ring system (Figure 2): 1,7‐naphthyridine from DB07204; 1,8‐naphthyridine from DB04903 (pagoclone), quinazoline from DB01157 (trimetrexate); isoquinoline from DB13931 (netarsudil). The workflow was composed as follow:

Step 1) The prepared library was imported in the KNIME workflow. The “RDKit Find Murcko Scaffold” node extracted the scaffold of each molecule. Then, the “group by” node created a collection of non‐redundant scaffold composed by 4791 scaffolds.

Step 2) Each scaffold was submitted to a fragmentation step by means the use of “MMP molecule fragment” node developed by RDKit. The scaffold was fragmented using the “Only acyclic single bonds to rings” fragmentation option, and the extracted fragments were converted to the corresponding cores using a second round of Murcko scaffold extraction performed on all the products of scaffold fragmentations (i. e. fragmentation key and fragmentation value). In total 9878 cores were collected.

Step 3) Among the collected cores the following filters were applied: number of ring=2; number of chiral centers=0. Additionally, only compounds with 6,6 bicyclic cores were retained using the “RDKit substructure filter” node (query SMART pattern: [*]1[*]2[*]([*][*][*]1)[*][*][*][*]2). The final quinoline‐like core‐hopping set was composed by 113 6,6 bicyclic cores.

The obtained cores were submitted to the core hopping utility within Schrodinger to generate the scaffold‐hopping library.^[49]^ The generated library was prepared within LigPrep. All the possible tautomeric and charged states at a pH range of 7.0±0.5 were produced and filtered using ADME properties calculated with QikProp.^[50]^ The ligands were filtered applying the following criteria: QPlogP oct/wat, from −2 to 6.5; QPPCaco≥500; QPlogKhsa, from −1.5 to 1.5; QPlogBB, from −3 to 1.2; Human oral absorption, ≥80 %; Polar surface area (PSA), ≤200; #stars≤1; rule of 5≤1; rule of three≤1; #chiral centers=0; Molecular charge=1; #metabolite ≤5. The remaining compounds were submitted to a conformational search using MacroModel^[51]^ setting the maximum number of steps to 10,000 per molecule. Conformers in an energy window of 5 kcal/mol were saved, discarding the redundant ones on the basis of their atomic rmsd (0.5 Å cutoff). Finally, the obtained conformers were screened in Phase^[35]^ using ModB and ModC as queries.

### Microbiological procedures

The *S. aureus* strains used in this work included SA‐1199 (wt), SA‐1199B (overexpressing *norA* and possessing an A116E GrlA substitution),^[52]^ SA‐K1902 (*norA*‐deleted) and its isogenic mutant SA‐K2378 overexpressing *norA*.^[53]^ All strains were cultured in Triptone Soy Agar or Mannitol Salt agar plates and maintained in Triptone Soy Broth supplemented with 20 % glycerol at −80 °C. Chloramphenicol (10 μg/mL) was added to the media when growing the strains SA‐K1902 and SA‐K2378. All culture media were purchased from Oxoid (Oxoid S.p.A., Rodano, Milano, Italy).

MICs assays were performed using broth microdilution method in 96 wells‐microtiter plates, following the CLSI guidelines;^[54]^ checkerboard assays were performed as previously described,^[38]^ using 2‐fold increasing concentrations of both antibiotic (from 0.02 to 20 μg/mL) and compounds (from 0.19 to 6.25 or 12.5 μg/mL) and considering combinations leading to a ≥4‐fold reduction of the CPX MIC as synergistic. All assays were performed in duplicate; when the results did not overlap, the higher MIC value was reported. EtBr efflux assays were performed as previously described^[44]^ and efflux activity was expressed as fluorescence decrease (%) over a 5‐minute time course. Efflux inhibition was determined using the equation ([efflux in the absence]−[efflux in the presence of test [compound])/[efflux in the absence of test compound]×100, giving the percent efflux inhibition observed. Assays were conducted in triplicate performing two technical replicates for each biological replicate. Time‐kill curves were performed as previously described,^[55]^ testing CPX concentrations ranging from 1/4 × to 1×MIC alone and in combination with the compound at 0.78 μg/mL. The dynamic of the bactericidal activity of the combination CPX‐Compound was evaluated by CFU counts after 2, 4, 6, 8 and 24 h incubation at 37 °C. Assays were performed testing at each time points 4 dilutions in duplicate.

### Cytotoxicity assays

The human monocytes cell line THP‐1 was purchased from ATCC (American Type Culture Collection), while the A549 (CCL‐185TM) cell line was kindly provided by Dr. Tatiana Armeni (Polytechnic University of Marche, Ancona, Italy). Cells were maintained respectively in Roswell Park Memorial Institute (RPMI‐1640) or in a 50 : 50 mixture of Dulbecco's Modified Eagle's Medium (DMEM) and Ham's F12 medium (F12), both from Corning Incorporated (Corning, New York, NY, USA), supplemented with 10 % fetal bovine serum (FBS, Corning Incorporated), 1 % L‐glutamine and 2 % antimycotic/antibiotic, at 37 °C in a humid atmosphere containing 5 % CO_2_. All antibiotics were purchased from Sigma‐Aldrich (Saint Louis, MO, USA).

The cytotoxic effect of the compounds was determined by MTT assays performed on THP‐1 and A549 (CCL‐185TM) cells after 24 h exposure.^[56]^ Cells were seeded at the density of 1×10^4^ cells/well into 96‐well flat‐bottomed plates in 200 μL of RPMI medium and then exposed for 24 h to the compounds at the concentrations resulted synergistic in checkerboard assays. Unexposed cells were used as negative control. The colorimetric MTT assay allowed to measure the cell growth rates through the amount of the accumulated intracellular insoluble formazan crystals, subsequently dissolved using DMSO and quantified spectrophotometrically (OD_540_), using a microplate reader (Neo Biotech NB‐12‐0035). The percentage of viable cells was calculated as follows: % Cell viability=100×Experimental well absorbance/untreated control well absorbance. All assays were performed in biological and technical duplicate.

### Chemistry

All starting materials, reagents and solvents were purchased from common commercial suppliers and were used as such, unless otherwise indicated. Organic solutions were dried over anhydrous Na_2_SO_4_ and concentrated with a rotary evaporator at low pressure. The reactions carried out under MW irradiation were performed employing a microwave reactor BIOTAGE INITIATOR 2.0 version 2.3, build 6250. All reactions were routinely checked by thin‐layer chromatography (TLC) on silica gel 60_F254_ (Merck) and visualized by using UV or iodine. Flash chromatography separations were carried out on Merck silica gel 60 (mesh 230–400) or by BUCHI Reveleris® X2 Flash Chromatography. Melting points were determined in capillary tubes (Stuart SMP30) and are uncorrected. Yields were of purified products and were not optimized. ^1^H NMR spectra were recorded at 200 or 400 MHz (Bruker Avance DRX‐200 or 400, respectively) while ^13^C NMR spectra were recorded at 101 MHz (Bruker Avance DRX‐400). Chemical shifts are given in ppm (*δ*) relative to TMS. Spectra were acquired at 298 K. Data processing was performed with standard Bruker software XwinNMR and the spectral data are consistent with the assigned structures. The purity of the tested compounds (≥95 % sample purity) was evaluated by HPLC analysis using a Jasco LC‐4000 instrument equipped with a UV‐Visible Diode Array Jasco MD‐4015 and an XTerra MS C18 Column, 5 μm, 4.6 mm×150 mm. Chromatograms were analyzed by ChromNAV 2.0 Chromatography Data System software.

**General procedure (A) for the synthesis of compounds 19–22**. Under N_2_ atmosphere, to a solution of derivatives **15**–**17** (1.0 equiv) in dry THF (4 mL per mmol), Et_3_N (3.0 or 5.0 equiv) was added. After stirring for 10 min, a suspension of properly substituted acyl chloride (1.0 equiv) in dry THF (3 mL per mmol) was dripped. The reaction mixture was stirred at room temperature or 60 °C for 3 h. The reaction mixture was then poured in ice/water and the obtained precipitate was filtered and then purified as described below.

**General procedure (B) for the synthesis of compounds 24–27**. Under N_2_ atmosphere, to a suspension of derivatives **19**–**22** (1.0 equiv) in *t*BuOH (5 mL per mmol), *t*BuOK (4.0 or 5.0 equiv) was added. The reaction mixture was stirred at room temperature or 90 °C for 90 min or 3 h and then poured in ice/water, modifying the pH up to 7 with 2 N HCl. Target compounds were recovered after filtration of the precipitate obtained or following an extraction with CH_2_Cl_2_ (3×100 mL).

**General procedure (C) for the synthesis of compounds 6 a**, **6 b**, **8 c and 8 d**. In a MW vial and under N_2_ atmosphere, derivatives **23** and **25** (1 equiv), chloroalkylamine (2.0 equiv) and K_2_CO_3_ (4.0 or 5.0 equiv) in dry DMF (3 mL per mmol) were added. The reaction mixture was irradiated by MW at the following conditions: time 10 or 15 min, max pressure 6 bar, cooling ON, temperature 90 or 100 °C. The mixture was then poured in ice/water and extracted with EtOAc (3×100 mL), the organic layer was washed with H_2_O (×6), then with brine, dried over Na_2_SO_4_ and evaporated to dryness to obtain a residue that was purified by flash column chromatography.

**General procedure (D) for the synthesis of compounds 8 a**, **8 b**, **8 e**, **11 a**, **11 b**, **12 a‐12 c**, **13 a** and **13 b**. Under N_2_ atmosphere, to a solution of derivatives **24**–**27** (1.0 equiv) in dry DMF (10 mL per mmol), K_2_CO_3_ (4.0 equiv), and chloroalkylamine (2.0 or 4.0 equiv) were added. The reaction mixture was stirred at 80 or 90 °C for 1 or 5 h and then poured in ice/water. Target compounds were recovered after filtration of the precipitate obtained or following an extraction with EtOAc or CH_2_Cl_2_ (3×100 mL). Reaction crudes were purified by flash column chromatography to give the title compounds.

***N*** **‐(4‐acetylpyridin‐3‐yl)‐4‐propoxybenzamide** (**18**). In a MW vial, the 1‐(3‐aminopyridin‐4‐yl)ethanone **14** (0.05 g, 0.37 mmol), DMAP (0.23 g, 1.85 mmol) and a solution of 4‐propoxybenzoyl chloride (0.30 g, 1.48 mmol) in dry dioxane (3 mL) were added. The reaction mixture was irradiated by MW at the following conditions: time 10 min, max pressure 6 bar, cooling ON, temperature 100 °C. Then, the mixture was poured in ice/water and extracted with CH_2_Cl_2_ (×3). The organic layer was washed with brine, dried over Na_2_SO_4_ and evaporated to dryness to give a brown oil. After purification by flash chromatography column (CH_2_Cl_2_/MeOH 97/3), compound **18** was obtained as a solid in 43 % yield (0.05 g). ^1^H NMR (200 MHz, DMSO‐*d_6_*,): δ_H_ 11.98 (s, 1H, NH), 10.21 (s, 1H, H2’), 8.44 (d, *J*=5.2 Hz, 1H, H6’), 7.91 (d, *J*=6.9 Hz, 2H, H2 and H6), 7.62 (d, *J*=5.1 Hz, 1H, H5’), 6.89 (d, *J*=8.9 Hz, 2H, H3 and H5), 3.79 (d, *J*=5.2 Hz, 2H, O*CH_2_* CH_2_CH_3_), 2.67 (s, 3H, CH_3_), 1.60–1.83 (m, 2H, OCH_2_ *CH_2_* CH_3_), 0.98 ppm (t, *J*=7.4 Hz, 3H, OCH_2_CH_2_ *CH_3_*).

***N‐*** **(3‐acetylpyridin‐2‐yl)‐4‐propoxybenzamide** (**19**). General procedure A (time=3 h, room temperature): starting from derivative **15** (0.15 g, 1.11 mmol) and using 4‐propoxybenzoyl chloride (0.22 g, 1.11 mmol) and Et_3_N (0.77 mL, 5.55 mmol), compound **19** was obtained, after trituration with a mixture of cyclohexane/EtOAc, as a white solid in 69 % yield (0.20 g). ^1^H NMR (400 MHz, CDCl_3_): δ_H_ 12.16 (s, 1H, NH), 8.75–8.70 (m, 1H, H6’), 8.20 (d, *J*=8.1 Hz,1H, H4’), 7.98 (d, *J*=8.6 Hz, 2H, H2 and H6), 7.15–7.10 (m, 1H, H5’), 6.88 (d, *J*=8.5 Hz, 2H, H3 and H5), 3.96 (t, *J*=7.5 Hz, 2H, O*CH_2_* CH_2_CH_3_), 2.45 (s, 3H, *CH_3_*), 1.82–1.65 (m, 2H, OCH_2_ *CH_2_* CH_3_), 1.04 ppm (t, *J*=7.4 Hz, 3H, OCH_2_CH_2_ *CH_3_*).

***N*** **‐[2‐(aminocarbonyl)phenyl]‐4‐propoxybenzamide (20)**. General procedure A (time=3 h, 60 °C): starting from derivative **16** (0.70 g, 5.00 mmol) and using 4‐propoxybenzoyl chloride (0.99 g, 5.00 mmol) and Et_3_N (3.4 mL, 25.0 mmol), compound **20** was obtained, after trituration with a mixture of CH_2_Cl_2_/MeOH, as a white solid in 71 % yield (1.00 g). ^1^H NMR (400 MHz DMSO‐*d*_6_): δ_H_ 12.73 (s, 1H, NH), 8.63 (d, *J*=8.4 Hz, 1H, H6’), 8.39 (s, 1H, NH_2_), 7.81 (d, *J*=5.5 Hz, 2H, H3’), 7.80 (d, *J*=8.7 Hz, 2H, H2 and H6), 7.51 (t, *J*=7.9 Hz, 1H, H4’), 7.11 (t, *J*=7.6 Hz, 1H, H5’), 7.03 (d, *J*=8.7 Hz, 2H, H3 and H5), 3.97 (t, *J*=6.5 Hz, 2H, O*CH_2_* CH_2_CH_3_), 1.75–1.67 (m, 2H, OCH_2_ *CH_2_* CH_3_), 0.94 ppm (t, *J=* 7.4 Hz, 3H, OCH_2_CH_2_ *CH_3_*).

**5‐Methoxy‐2‐[(4‐propoxybenzoyl)amino]benzamide** (**21**). General procedure A (time=3 h, 60 °C): starting from derivative **17** (0.50 g, 3.00 mmol) and using 4‐propoxybenzoyl chloride (0.59 g, 3.00 mmol) and Et_3_N (1.25 mL, 9.00 mmol), compound **21** was obtained, after trituration with Et_2_O, as a white solid in 71 % yield (0.36 g). ^1^H NMR (400 MHz, DMSO‐*d*_6_): δ_H_ 12.34 (1H, s, NH), 8.54 (1H, d, *J*=9.1 Hz, H3), 8.37 (s, 1H, NH_2_), 7.80 (d, *J*=8.8 Hz, 2H, H2’ and H6’), 7.34 (d, *J*=2.6 Hz, 1H, H6), 7.11 (dd, *J*=2.8 and 9.2 Hz, 1H, H4), 7.03 (d, *J*=8.7 Hz, 2H, H3’ and H5’), 3.95 (t, *J*=6.5 Hz, 2H, O*CH_2_* CH_2_CH_3_), 3.74, (s, 3H, CH_3_), 1.73–1.65 (m, 2H, OCH_2_ *CH_2_* CH_3_), 0.93 ppm (t, *J*=7.4 Hz, 3H, OCH_2_CH_2_ *CH_3_*).

***N*** **‐[2‐(aminocarbonyl)‐4‐methoxyphenyl]‐5‐chlorothiophene‐2‐carboxamide (22)**. General procedure A (time=3 h, 60 °C): starting from derivative **17** (2.52 g, 15.0 mmol) and using 5‐chlorothiophene‐2‐carbonyl chloride (2.44 g, 15.0 mmol) and Et_3_N (6.25 mL, 45.0 mmol), compound **22** was obtained, after trituration with Et_2_O, as white solid in 40 % yield (1.86 g). ^1^H NMR (400 MHz, DMSO‐*d*_6_): δ_H_ 12.70 (s, 1H, NH), 8.40 (s, 1H, 1/2 NH_2_), 8.34 (d, *J*=9.1 Hz, 1H, H6’), 7.84 (s, 1H, 1/2 NH_2_), 7.48 (d, *J*=4.2 Hz, 1H, H3), 7.39 (d, *J*=2.9 Hz, 1H, H3’), 7.23 (d, *J*=4.5 Hz, 1H, H4), 7.14–7.11 (m, 1H, H5’), 3.79 ppm (s, 3H, OCH_3_).

**2‐(4‐Propoxyphenyl)‐1,7‐naphthyridin‐4‐ol** (**23**). In a MW vial and under N_2_ atmosphere, *N*‐(4‐acetylpyridin‐3‐yl)‐4‐propoxybenzamide **18** (0.10 g, 0.34 mmol), NaOH (0.04 g, 1.01 mmol) and dry dioxane (1.5 mL) were added. The reaction mixture was irradiated by MW at the following conditions: time 10 min, max pressure 6 bar, cooling ON, temperature 110 °C. The reaction mixture was poured in ice/water modifying the pH up to 7 with 2 N HCl and extracted with CH_2_Cl_2_ (×3). The organic layer was washed with brine, dried over Na_2_SO_4_ and evaporated to dryness to give a yellow solid. After purification by flash chromatography column (CH_2_Cl_2_/MeOH 97/3), compound **23** was obtained as a withe solid in 40 % yield (0.04 g). ^1^H NMR (400 MHz, DMSO‐*d_6_*): δ_H_ 11.92 (s, 1H, OH), 9.14 (s, 1H, H8), 8.42 (d, *J*=5.3 Hz, 1H, H6), 7.86 (d. *J*=5.9 Hz, 1H, H5), 7.81 (d, *J*=8.6 Hz, 2H, H2’ and H6’), 7.10 (d, *J*=8.9 Hz, 2H, H3’ and H5’), 6.46 (s, 1H, H3), 4.00 (t, *J*=6.5 Hz, 2H, O*CH_2_* CH_2_CH_3_), 1.81–1.65 (m, 2H, OCH_2_ *CH_2_* CH_3_), 0.96 ppm (t, *J*=7.4 Hz, 3H, OCH_2_CH_2_ *CH_3_*).

**2‐(4‐Propoxyphenyl)‐1,8‐naphthyridin‐4‐ol** (**24**). General procedure B (time=90 min, 90 °C): starting from derivative **19** (0.10 g, 0.34 mmol) and using *t*BuOK (0.19 g, 1.70 mmol), compound **24** was obtained, after extraction with CH_2_Cl_2_, as a yellow solid in 87 % yield (0.08). ^1^H NMR (400 MHz, CDCl_3_): δ_H_ 10.47 (s, 1H, OH), 8.68–8.60 (m, 1H, H7), 8.26–8.23 (m, 1H, H5), 7.58 (d, *J*=8.8 Hz, 2H, H2’ and H6’), 7.25–7.23 (m, 1H, H6), 6.89 (d, *J*=8.7 Hz, 2H, H3’ and H5’), 6.50 (s, 1H, H3), 3.95 (t, *J*=7.5 Hz, 2H, O*CH_2_* CH_2_CH_3_), 1.82–1.65 (m, 2H, OCH_2_ *CH_2_* CH_3_), 1.04 ppm (t, *J*=7.4 Hz, 3H, OCH_2_CH_2_ *CH_3_*).

**2‐(4‐Propoxyphenyl)quinazolin‐4‐ol (25)**.^[41]^ General procedure B (time=3 h, room temperature): starting from derivative **20** (2.23 g, 7.47 mmol) and using *t*BuOK (3.35 g, 29.80 mmol), compound **25** was obtained, after extraction with CH_2_Cl_2_, as a yellow solid in 86 % yield (1.80 g). ^1^H NMR (400 MHz, DMSO‐*d*_6_): δ_H_ 12.35 (s, 1H, OH), 8.14 (d, *J*=8.6 Hz, 2H, H2’ and H6’), 8.09 (d, *J*=7.8 Hz, 1H, H8), 7.76 (t, *J*=7.5 Hz, 1H, H7), 7.65 (d, *J*=8.1 Hz, 1H, H5), 7.43 (t, *J*=7.4 Hz, 1H, H6), 7.03 (d, *J*=8.6 Hz, 2H, H3’ and H5’), 3.97 (t, *J*=6.5 Hz, 2H, O*CH_2_* CH_2_CH_3_), 1.76–1.72 (m, 2H, OCH_2_ *CH_2_* CH_3_), 0.94 ppm (t, *J*=7.4 Hz, 3H, OCH_2_CH_2_ *CH_3_*).

**6‐Methoxy‐2‐(4‐propoxyphenyl)quinazolin‐4‐ol** (**26**). General procedure B (time=3 h, room temperature): starting from derivative **21** (0.61 g, 1.82 mmol) and using *t*BuOK (0.81 g, 7.28 mmol), compound **26** was obtained, after filtration, as a white solid in 90 % yield (0.50 g). ^1^H NMR (400 MHz, DMSO‐*d*_6_): δ_H_ 12.34 (s, 1H, OH), 8.10 (d, *J*=8.8 Hz, 2H, H2’ and H6’), 7.60 (d, *J*=8.9 Hz, 1H, H8), 7.48 (d, *J*=2.9 Hz, 1H, H5), 7.36 (dd, *J*=2.8 and 9.2 Hz, 1H, H7), 7.01 (d, *J*=8.7 Hz, 2H, H3’ and H5’), 3.97 (t, *J*=6.5 Hz, 2H, O*CH_2_* CH_2_CH_3_), 3.84, (s, 3H, CH_3_), 1.72–1.67 (m, 2H, OCH_2_ *CH_2_* CH_3_), 0.95 ppm (t, *J*=7.4 Hz, 3H, OCH_2_CH_2_ *CH_3_*).

**2‐(5‐Chloro‐2‐thienyl)‐6‐methoxyquinazolin‐4‐ol** (**27**). General procedure B (time=3 h, room temperature): starting from derivative **22** (0.60 g, 1.93 mmol) and using *t*BuOK (0.86 g, 7.72 mmol), compound **27** was obtained, after filtration, as a white solid in 75 % yield (0.40 g). ^1^H NMR (400 MHz, DMSO‐*d*_6_): δ_H_ 12.67 (s, 1H, OH), 8.02 (d, *J*=3.9 Hz, 1H, H4’), 7.61 (d, *J*=8.9 Hz, 1H, H8), 7.57 (d, *J*=2.5 Hz, 1H, H5), 7.39 (dd, *J*=2.7 and 7.6 Hz, 1H, H7), 7.24 (d, *J*=3.9 Hz, 1H, H3’), 3.88 ppm (s, 3H, OCH_3_).

**4‐Chloro‐2‐(4‐propoxyphenyl)‐1,8‐naphthyridine (28)**. At 0 °C, POCl_3_ (1.5 mL) was added to 2‐(4‐propoxyphenyl)‐1,8‐naphthyridin‐4‐ol **24** (0.10 g, 0.36 mmol) and then the mixture was stirred at 100 °C for 3 h. Then, the excess of POCl_3_ was evaporated and the sat. sol. of NaHCO_3_ was added to the reaction mixture at 0 °C. After stirring overnight, the obtained yellow precipitate was filtered to give compound **28** in 80 % yield (0.08 g). ^1^H NMR (400 MHz, DMSO‐*d*_6_): δ_H_ 9.14 (dd, *J*=1.8 and 8.4 Hz, 1H, H7), 8.66 (dd, *J*=1.7 and 8.3 Hz, 1H, H5), 8.51 (s,1H, H3), 8.31 (d, *J*=8.9 Hz, 2H, H2’ and H6’), 7.75–7.72 (m,1H, H6), 7.09 (d, *J*=8.9 Hz, 2H, H3’ and H5’), 4.01 (t, *J*=6.6 Hz, 2H, O*CH_2_* CH_2_CH_3_), 1.78–1.69 (m, 2H, OCH_2_ *CH_2_* CH_3_), 0.97 ppm (t, *J*=7.4 Hz, 3H, OCH_2_CH_2_ *CH_3_*).

***N***, ***N*** **‐diethyl‐2‐{[2‐(4‐propoxyphenyl)‐1,7‐naphthyridin‐4‐yl]oxy}ethanamine (6 a)**. General procedure C (time 20 min. 100 °C): starting from derivative **23** (0.20 g, 0.71 mmol) and using (2‐chloroethyl)diethylamine hydrochloride (0.24 g, 1.42 mmol) and K_2_CO_3_ (0.39 g, 2.84 mmol), compound **6 a** was obtained, after purification via flash chromatography column (CH_2_Cl_2_/MeOH 95 : 5), as a pink solid in 15 % yield (0.04 g); mp=69.0–69.5 °C ^1^H NMR (400 MHz, CDCl_3_): δ_H_ 9.41 (s,1H, H8), 8.49 (d, *J*=5.6 Hz, 1H, H6), 8.05 (d, *J*=8.4 Hz, 2H, H2’ and H6’), 7.87 (d, *J*=5.3 Hz, 1H, H5), 7.26 (s, 1H, H3), 7.00 (d, *J*=8.4 Hz, 2H, H3’ and H5’), 4.31 (t, *J*=5.8 Hz, 2H, O*CH_2_* CH_2_N), 3.97 (t, *J*=6.5 Hz, 2H, O*CH_2_* CH_2_CH_3_), 3.04 (t, *J*=5.7 Hz, 2H, OCH_2_ *CH_2_* N), 2.68 (q, *J*=7.0 Hz, 4H, N*CH_2_* CH_3_×2), 1.85–1.80 (m, 2H, OCH_2_ *CH_2_* CH_3_), 1.10 (t, *J*=7.0 Hz, 6H, NCH_2_ *CH_3_* ×2), 1.04 ppm (t, *J*=7.3 Hz, 3H, OCH_2_CH_2_ *CH_3_*).^13^C NMR (101 MHz, CDCl_3_): δ_C_ 161.01, 160.76, 159.82, 153.37, 144.16, 142.56, 131.65, 128.85, 123.89, 114.75, 114.38, 100.99, 69.62, 67.79, 51.26, 48.10, 22.54, 12.10, 10.52 ppm. HPLC: 70 % CH_3_CN, 30 % H_2_O/0.1 Et_2_NH, rt=6.03 min.

**4‐(2‐Piperidin‐1‐ylethoxy)‐2‐(4‐propoxyphenyl)‐1,7‐naphthyridine (6 b)**. General procedure C (time 10 min. 100 °C): starting from derivative **23** (0.20 g, 0.71 mmol) and using 1‐(2‐chloroethyl)piperidine hydrochloride (0.26 g, 1.42 mmol) and K_2_CO_3_ (0.39 g, 2.84 mmol), compound **6 b** was obtained, after purification via flash chromatography column (CH_2_Cl_2_/MeOH 95 : 5), as a pink solid in 14 % yield (0.04 g); mp=92.5–94.0 °C. ^1^H NMR (400 MHz, CDCl_3_,): δ_H_ 9.41 (s, 1H, H8), 8.49 (d, *J*=5.5 Hz, 1H, H6), 8.05 (d, *J*=8.6 Hz, 2H, H2’ and H6’), 7.86 (d, *J*=5.5 Hz, 1H, H5), 7.25 (s, 1H, H3), 7.00 (d, *J*=8.6 Hz, 2H, H3’ and H5’), 4.38 (t, *J*=5.9 Hz, 2H, O*CH_2_* CH_2_N), 3.97 (t, *J*=6.5 Hz, 2H, O*CH_2_* CH_2_CH_3_), 2.94 (t, *J*=5.8 Hz, 2H, OCH_2_ *CH_2_* N), 2.61–2.52 (m, 4H, piperidine N*CH_2_* ×2), 1.87–1.78 (m, 2H, OCH_2_ *CH_2_* CH_3_), 1.63–1.58 (m, 4H, piperidine CH_2_×2), 1.45–1.44 (m, 2H, piperidine CH_2_), 1.04 ppm (t, *J*=7.4 Hz, 3H, OCH_2_CH_2_ *CH_3_*). ^3^C NMR (101 MHz, CDCl_3_): δ_C_ 160.90, 160.78, 159.82, 153.37, 144.17, 142.58, 131.66, 128.85, 123.89, 114.77, 114.36, 101.01, 69.63, 67.11, 57.43, 55.18, 26.00, 24.04, 22.54, 10.50 ppm. HPLC: 80 % CH_3_CN, 20 % H_2_O/0.1 Et_2_NH, rt=4.67 min.

**4‐(2‐Piperidin‐1‐ylethoxy)‐2‐(4‐propoxyphenyl)‐1,8‐naphthyridine (7 b)**. Under N_2_ atmosphere, to a suspension of 60 % NaH (0.04 g, 0.66 mmol) in dry DMF (5 mL), 1‐(2‐hydroxyethyl)piperidine (0.06 mL, 0.66 mmol) was added. After stirring for 10 min at room temperature, 4‐chloro‐2‐(4‐propoxyphenyl)‐1,8‐naphthyridine **28** (0.05 g, 0.16 mmol) was added and the reaction mixture was stirred at room temperature for 3 h. Then, the excess of NaH was quenched with EtOAc and the mixture was poured in ice/water and extracted with EtOAc (x3). The organic layer was washed with brine, dried over Na_2_SO_4_ and evaporated to dryness to give a yellow oil. After purification by flash chromatography column (CH_2_Cl_2_/MeOH 97/3), compound **7 b** was obtained as a brown solid in 41 % yield (0.08 g); mp=142.0–142.5 °C. ^1^H NMR (400 MHz, CDCl_3_): δ_H_ 9.00 (dd, *J*=1.9 and 7.2 Hz, 1H, H7), 8.46 (dd, *J*=1.9 and 8.1 Hz, 1H, H5), 8.19 (d, *J*=8.7 Hz,2H, H2’ and H6’), 7.35–7.32 (m, 1H, H6), 7.22 (s, 1H, H3), 6.98 (d, *J*=8.7 Hz, 2H, H3’ and H5’), 4.39 (t, *J*=5.9 Hz, 2H, O*CH_2_* CH_2_N), 3.97 (t, *J*=6.5 Hz, 2H, O*CH_2_* CH_2_CH_3_), 2.94 (t, *J*=5.9 Hz, 2H, OCH_2_ *CH_2_* N), 2.59–2.51 (m, 4H, piperidine NCH_2_×2), 1.88–1.77 (m, 2H, OCH_2_ *CH_2_* CH_3_), 1.64–1.58 (m, 4H, piperidine CH_2_×2), 1.47–1.43 (m, 2H, piperidine CH_2_) 1.03 ppm (t, *J*=7.4 Hz, 3H, OCH_2_CH_2_ *CH_3_*). ^13^C NMR (101 MHz, CDCl_3_): δ_C_ 162.30, 160.93, 160.87, 157.22, 153.63, 131.53, 131.40, 129.22, 120.28, 114.91, 114.50, 98.38, 69.57, 67.17, 57.49, 55.17, 25.99, 24.05, 22.57, 10.53 ppm. HPLC: 70 % CH_3_CN, 30 % H_2_O/0.1 Et_2_NH, rt=4.18 min.

***N***, ***N*** **‐diethyl‐2‐{[2‐(4‐propoxyphenyl)quinazolin‐4‐yl]oxy}ethanamine hydrochloride (8 a)**. General procedure D (time=1 h): starting from derivative **25** (0.40 g, 1.30 mmol) and using K_2_CO_3_ (0.72 g, 5.20 mmol) and (2‐chloroethyl)diethylamine hydrochloride (0.45 g, 2.60 mmol), the free base of compound **8 a** was obtained, after extraction with EtOAc and purification by flash chromatography column (EtOAc/MeOH 95/5), as a yellow oil in 11 % yield (0.05 g). HCl_gas_ was bubbled into a solution of the oil in Et_2_O until obtaining a precipitate that was recovered by filtration to give compound **8 a** as a yellow solid; mp=183.5–184.0 °C. ^1^H NMR (400 MHz, DMSO‐*d*_6_): δ_H_ 10.85 (s,1H, HCl), 8.49 (d, *J*=8.8 Hz, 2H, H2’ and H6’), 8.25 (d, *J*=8.1 Hz, 1H, H5), 8.02 (d, *J*=8.3 Hz, 1H, H8), 7.95 (t, *J*=7.2 Hz, 1H, H6), 7.64 (t, *J*=7.3 Hz, 1H, H7), 7.09 (d, *J*=8.9 Hz, 2H, H3’ and H5’), 5.05 (t, *J*=4.1 Hz, 2H, O*CH_2_* CH_2_N), 3.97 (t, *J*=6.5 Hz, 2H, O*CH_2_* CH_2_CH_3_), 3.59 (d, *J*=5.9 Hz, 2H, OCH_2_ *CH_2_* N), 3.30–3.20 (m, 4H, N*CH_2_* CH_3_×2), 1.78–1.69 (m, 2H, CH_2_ *CH_2_* CH_3_), 1.27 (t, *J* =7.2 Hz, 6H, NCH_2_ *CH_3_* ×2), 0.97 ppm (t, *J*=7.4 Hz, 3H, OCH_2_CH_2_ *CH_3_*). ^13^C NMR (101 MHz, DMSO‐*d*_6_): δ_C_ 166.30, 161.99, 158.97, 150.46, 135.30, 130.70, 128.80, 127.56, 126.76, 124.26, 114.92, 114.51, 69.66, 62.04, 49.68, 47.37, 22.46, 10.86, 8.98 ppm. HPLC: 90 % CH_3_CN 10 %, H_2_O/0.1 Et_2_NH, rt=4.61 min.

**4‐(2‐Piperidin‐1‐ylethoxy)‐2‐(4‐propoxyphenyl)quinazoline (8 b)**. General procedure D (time=1 h): starting from derivative **25** (0.50 g, 1.62 mmol) and using K_2_CO_3_ (0.89 g, 6.48 mmol) and 1‐(2‐chloroethyl)piperidine hydrochloride (0.59 g, 3.24 mmol), compound **8 b** was obtained, after extraction with EtOAc and purification by flash chromatography column (EtOAc/MeOH 95/5), as yellow solid in 17 % yield (0.10 g); mp=84.0–84.5 °C . ^1^H NMR (400 MHz, CDCl_3_): δ_H_ 8.49 (d, *J*=9.0 Hz, 2H, H2’ and H6’), 8.09 (dd, *J*=1.8 and 8.2 Hz, 1H, H5), 7.91 (d, *J*=8.3 Hz, 1H, H8), 7.76 (dt, *J*=1.5 and 8.4 Hz, 1H, H7), 7.44 (dt, *J*=1.7 and 8.1 Hz, 1H, H6), 6.98 (d, *J*=9.0 Hz, 2H, H3’ and H5’), 4.87 (t, *J*=5.9 Hz, 2H, O*CH_2_* CH_2_N), 3.99 (t, *J*=6.6 Hz, 2H, O*CH_2_* CH_2_CH_3_), 2.99 (t, *J*=5.8 Hz, 2H, OCH_2_ *CH_2_* N), 2.69–2.60 (m, 4H, piperidine N*CH_2_* ×2), 1.87–1.79 (m, 2H, OCH_2_ *CH_2_* CH_3_), 1.67–1.64 (m, 4H, piperidine CH_2_×2), 1.47–1.46 (m, 2H, piperidine CH_2_), 1.05 ppm (t, *J*=7.4 Hz, 3H, OCH_2_CH_2_ *CH_3_*).^13^C NMR (CDCl_3_, 101 MHz): δ_C_ 166.19, 161.23, 159.78, 151.97, 133.35, 130.42, 129.97, 127.63, 125.84, 123.36, 114.90, 114.19, 69.50, 64.23, 57.26, 54.85, 25.62, 23.86, 22.51, 10.46 ppm. HPLC: 80 % CH_3_CN, 20 % H_2_O/0.1 Et_2_NH, rt=12.02 min.

**4‐(2‐Azepan‐1‐ylethoxy)‐2‐(4‐propoxyphenyl)quinazoline (8 c)**. General procedure C (time 15 min. 90 °C): starting from derivative **25** (0.10 g, 0.32 mmol) and using 1‐(2‐chloroethyl)azepane hydrochloride (0.13 g, 0.64 mmol) and K_2_CO_3_ (0.22 g, 1.60 mmol), compound **8 c** was obtained, after purification via flash chromatography column (EtOAc/MeOH 97/3), as a white solid in 84 % yield (0.10 g); mp =112.5–113.0 °C . ^1^H NMR (400 MHz, CDCl_3_): δ_H_ 8.50 (d, *J*=8.9 Hz, 2H, H2’ and H6’), 8.10 (d, *J*=8.5 Hz, 1H, H5), 7.91 (d, *J*=8.5 Hz, 1H, H8), 7.78–7.74 (m, 1H, H7), 7.46–7.43 (m, 1H, H6), 6.99 (d, *J*=8.9 Hz, 2H, H3’ and H5’), 4.79 (t, *J*=5.0 Hz, 2H, O*CH_2_* CH_2_N), 3.99 (t, *J*=6.5 Hz, 2H, O*CH_2_* CH_2_CH_3_), 3.11 (t, *J*=6.3 Hz, 2H, OCH_2_ *CH_2_* N), 2.89–2.80 (m, 4H, azepane NCH_2_×2), 1.88–1.79 (m, 2H, OCH_2_ *CH_2_* CH_3_), 1.67–1.59 (m, 8H, azepane CH_2_×4), 1.05 ppm (t, *J*=7.4 Hz, 3H, OCH_2_CH_2_ *CH_3_*). ^13^C NMR (101 MHz, CDCl_3_): δ_C_ 166.42. 161.24, 159.84, 151.96, 133.27, 130.54, 129.96, 127.60, 125.76, 123.43, 115.01, 114.18, 69.50, 65.02, 55.98, 55.77, 28.11, 26.98, 22.51, 10.46 ppm. HPLC: 80 % CH_3_CN, 20 % H_2_O/0.1 Et_2_NH, rt=17.74 min.

***N***, ***N*** **‐dimethyl‐3‐{[2‐(4‐propoxyphenyl)quinazolin‐4‐yl]oxy}propan‐1‐amine (8 d)**. General procedure C (time 15 min. 90 °C): starting from derivative **25** (0.40 g, 1.30 mmol) and using (3‐chloropropyl)dimethylamine hydrochloride (0.41 g, 2.60 mmol) and K_2_CO_3_ (0.89 g, 6.50 mmol), compound **8 d** was obtained, after purification via flash chromatography column (CH_2_Cl_2_ /MeOH 95/5 to 90/10), as a yellow solid in 39 % yield (0.20 g); mp=71.5–73.0 °C. ^1^H NMR (400 MHz, CDCl_3_): δ_H_ 8.50 (d, *J*=8.8 Hz, 2H, H2’ and H6’), 8.12–8.07 (m, 1H, H5), 7.92–7.90 (m, 1H, H8), 7.78–7.74 (m, 1H, H7), 7.47–7.43 (m, 1H, H6), 6.98 (d, *J*=8.9 Hz, 2H, H3’ and H5’), 4.73 (t, *J*=5.0 Hz, 2H, O*CH_2_* CH_2_CH_2_N), 3.96 (t, *J*=6.5 Hz, 2H, O*CH_2_* CH_2_CH_3_), 2.57–2.54 (m, 2H, OCH_2_CH_2_ *CH_2_* N), 2.39 (s, 6H, NCH_3_×2), 2.14–2.06 (m, 2H, OCH_2_ *CH_2_* CH_2_N), 1.86–1.79 (m, 2H, OCH_2_ *CH_2_* CH_3_), 1.05 ppm (t, *J*=7.4 Hz, 3H, OCH_2_CH_2_ *CH_3_*). ^13^C NMR (101 MHz, CDCl_3_): δ_C:_ 166.42, 161.23, 159.86, 151.93, 133.27, 130.55, 129.97, 127.63, 125.75, 123.34, 114.96, 114.17, 69.49, 64.98, 56.47, 45.41, 27.02, 22.51, 10.46 ppm. HPLC: 80 % CH_3_CN, 20 % H_2_O/0.1 Et_2_NH, rt=8.54 min.

**4‐[2‐(4‐Benzylpiperazin‐1‐yl)ethoxy]‐2‐(4‐propoxyphenyl)quinazoline hydrochloride (8 e)**. General procedure D (time=3 h 90 °C): starting from derivative **25** (0.20 g, 0.71 mmol) and using K_2_CO_3_ (0.49 g, 3.55 mmol) and 1‐benzyl‐4‐(2‐chloroethyl)piperazine (0.34 g, 1.43 mmol), the free base of compound **8 e** was obtained, after extraction with EtOAc and purification by flash chromatography column (CH_2_Cl_2_/MeOH 98/2), as a yellow oil in 40 % yield (0.10 g). HCl_gas_ was bubbled into a solution of the oil in Et_2_O until obtaining a precipitate that was recovered by filtration to give compound **8 e** as a white solid; mp =246.0–248.0 °C . ^1^H NMR (400 MHz, CDCl_3_): δ_H_ 8.48 (d, *J*=8.9 Hz, 2H, H2’ and H6’), 8.08 (dd, *J*=1.8 and 8.1 Hz, 1H, H5), 7.91 (d, *J*=8.3 Hz, 1H, H8), 7.76 (dt, *J*=1.7 and 8.4 Hz, 1H, H7), 7.44 (dt, *J*=1.7 and 8.1 Hz, 1H, H6), 7.31–7.24 (m, 5H, ArH), 6.98 (d, *J*=8.9 Hz, 2H, H3’ and H5’), 4.83 (t, *J*=5.7 Hz, 2H, O*CH_2_* CH_2_N), 3.99 (t, *J*=6.6 Hz, 2H, O*CH_2_* CH_2_CH_3_), 3.54–3.46 (m, 2H, N*CH_2_* Ph), 2.99 (t, *J*=5.6 Hz, 2H, OCH_2_ *CH_2_* N), 2.73–2.55 (m, 8H, piperazine CH_2_ x 4), 1.88–1.79 (m, 2H, OCH_2_ *CH_2_* CH_3_), 1.05 ppm (t, *J*=7.4 Hz, 3H, OCH_2_CH_2_ *CH_3_*).^13^C NMR (101 MHz, Acetone): 166.40, 161.46, 159.33, 151.93, 133.61, 130.33, 129.85, 128.73, 127.98, 127.53, 126.71, 126.10, 123.31, 114.77, 114.03, 69.18, 64.55, 62.50, 56.52, 53.50, 53.06, 22.27, 9.79 ppm. HPLC: 90 % CH_3_CN, 10 % H_2_O/0.1 Et_2_NH, rt=6.60 min.

**4‐(2‐Piperazin‐1‐ylethoxy)‐2‐(4‐propoxyphenyl)quinazoline (8 f)**. Under a N_2_ atmosphere, to a solution of 4‐[2‐(4‐benzylpiperazin‐1‐yl)ethoxy]‐2‐(4‐propoxyphenyl)quinazoline **8 e** (0.30 g, 0.60 mmol) in MeOH (15 mL), Pd/C (0.30 g) and ammonium formate (0.19 g, 3.02 mmol) were added. The reaction mixture was stirred at reflux for 2 h. Then, it was filtered over Celite and the filtrate was evaporated to dryness to give a yellow oil. After purification by flash chromatography column (CHCl_3_/MeOH 90/10), compound **8 f** was obtained as a white solid in 14 % yield (0.03 g); mp=178.0–179.5 °C. ^1^H NMR (400 MHz, DMSO‐*d*_6_): δ_H_ 8.92 (s, 1H, H5), 8.42 (d, *J*=8.7, 2H, H2’ and H6’), 8.08 (d, *J*=8.2 Hz, 1H, H8), 7.91–7.83 (m, 2H, H7), 7.99–7.95 (m, 1H, H6), 7.04 (d, *J*=8.7 Hz, 2H, H3’ and H5’), 4.78 (t, *J*=5.1 Hz, 2H, O*CH_2_* CH_2_N), 3.98 (t, *J*=6.5 Hz, 2H, O*CH_2_* CH_2_CH_3_), 3.29 (s, 1H, NH), 3.04–2.98 (m, 4H, piperazine CH_2_×2), 2.94 (t, *J*=5.1 Hz, 2H, OCH_2_ *CH_2_* N), 2.79–2.71 (m, 4H, piperazine CH_2_×2), 1.78–1.69 (m, 2H, OCH_2_ *CH_2_* CH_3_), 0.97 ppm (t, *J*=7.4 Hz, 3H, OCH_2_CH_2_ *CH_3_*). ^13^C NMR (101 MHz, DMSO‐*d*_6_): δ_C_ 166.48, 161.50, 159.26, 151.78, 134.67, 130.20, 130.09, 127.83, 127.10, 123.75, 114.83, 114.71, 69.58, 64.59, 56.14, 49.80, 43.27, 22.46, 10.84 ppm. HPLC: 90 % CH_3_CN, 10 % H_2_O/0.1 Et_2_NH, rt=4.66 min.

**1‐[2‐(Diethylamino)ethyl]‐2‐(4‐propoxyphenyl)‐1,8‐naphthyridin‐4(1*H*)‐one (11 a)**. General procedure D (time=3 h): starting from derivative **24** (0.30 g, 1.07 mmol) and using K_2_CO_3_ (0.59 g, 4.28 mmol) and (2‐chloroethyl)diethylamine hydrochloride (0.73 g, 4.28 mmol), compound **11 a** was obtained after filtration and purification by flash chromatography column (CH_2_Cl_2_/MeOH 93/7), as a yellow solid in 50 % yield (0.20 g); mp=194.0–194.5 °C. ^1^H NMR (400 MHz, CDCl_3_): δ_H_ 9.02 (dd, *J*=1.5 and 7.6 Hz, 1H, H7), 8.18 (dd, *J*=1.4 and 7.5 Hz, 1H, H5), 8.00–8.07 (m, 2H, H2’ and H6’), 7.03–6.99 (m, 2H, H3 and H6), 6.94 (d, *J*=8.2 Hz, 2H, H3’ and H5’), 4.75 (t, *J*=5.5 Hz, 2H, N*CH_2_* CH_2_N), 3.60 (t, *J*=6.6 Hz, 2H, O*CH_2_* CH_2_CH_3_), 2.96 (t, *J*=5.5 Hz, 2H, NCH_2_ *CH_2_* N), 2.48 (q, *J*=7.2 Hz, 4H, N*CH_2_* CH_3_×2), 1.86–1.77 (m, 4H, OCH_2_ *CH_2_* CH_3_), 1.03 (t, *J*=7.4 Hz, 3H, OCH_2_CH_2_ *CH_3_*), 0.79 ppm (t, *J*=7.2 Hz, 6H, NCH_2_ *CH_3_* ×2).^13^C NMR (101 MHz, CDCl_3_): δ_C_ 177.30, 160.91, 160.77, 150.74, 144.56, 142.64, 131.67, 128.90, 125.18, 114.38, 111.33, 108.61, 69.59, 52.93, 51.38, 47.75, 22.54, 12.23, 10.52 ppm. HPLC: 80 % CH_3_CN, 20 % H_2_O/0.1 Et_2_NH, rt=2.04 min.

**1‐(2‐Piperidin‐1‐ylethyl)‐2‐(4‐propoxyphenyl)‐1,8‐naphthyridin‐4(1*H*)‐one (11 b)**. General procedure D (time=3 h): starting from derivative **24** (0.30 g, 1.07 mmol) and using K_2_CO_3_ (0.59 g, 4.28 mmol) and 1‐(2‐chloroethyl)piperidine hydrochloride (0.80 g, 4.28 mmol), compound **11 b** was obtained after filtration and purification by flash chromatography column (CH_2_Cl_2_/MeOH 93/7), as a yellow solid in 66 % yield (0.30 g); mp=217.0–217.5 °C. ^1^H NMR (400 MHz, CDCl_3_): δ_H_ 8.99 (dd, *J*=1.5 and 7.6 Hz, 1H, H7), 8.18 (dd, *J*=1.4 and 7.5 Hz, 1H, H5), 8.03 (d, *J*=8.8 Hz, 2H, H2’ and H6’), 7.00–6.97 (m, 2H, H3 and H6), 6.94 (d, *J*=8.2 Hz, 2H, H3’ and H5’), 4.80 (t, *J*=5.8 Hz, 2H, N*CH_2_* CH_2_N), 3.96 (t, *J*=6.5 Hz, 2H, O*CH_2_* CH_2_CH_3_), 2.85 (t, *J*=5.8 Hz, 2H, NCH_2_ *CH_2_* N), 2.44–2.38 (m, 4H, piperidine NCH_2_×2), 1.85–1.76 (m, 2H, OCH_2_ *CH_2_* CH_3_), 1.49–1.48 (m, 4H, piperidine CH_2_×2), 1.47–1.41 (m, 2H, piperidine CH_2_), 1.02 ppm (t, *J*=7.4 Hz, 3H, OCH_2_CH_2_ *CH_3_*). ^13^C NMR (101 MHz, CDCl_3_): δ_C_ 177.23, 160.94, 160.79, 150.81, 144.26, 142.56, 131.67, 128.87, 125.26, 114.40, 111.51, 108.57, 69.60, 56.95, 54.72, 50.96, 26.03, 24.02, 22.54, 10.49 ppm. HPLC: 70 % CH_3_CN, 30 % H_2_O/0.1 Et_2_NH, rt=2.24 min.

***N***, ***N*** **‐diethyl‐2‐{[6‐methoxy‐2‐(4‐propoxyphenyl)quinazolin‐4‐yl]oxy}ethanamine (12 a)**. General procedure D (time=3 h): starting from derivative **26** (0.30 g, 0.97 mmol) and using K_2_CO_3_ (0.40 g, 2.91 mmol) and (2‐chloroethyl)diethylamine hydrochloride (0.33 g, 1.94 mmol), compound **12 a** was obtained, after filtration and purification by flash chromatography (EtOAc/MeOH 98/2 to 95/5), as a white solid in 18 % yield (0.70 g); mp=82.5–83.5 °C . ^1^H NMR (400 MHz, CDCl_3_): δ_H_ 8.45 (d, *J*=8.7 Hz, 2H, H2’ and H6’), 7.83 (d, *J*=9.0 Hz, 1H, H8), 7.41–7.36 (m, 2H, H5 and H7), 6.97 (d, *J*=8.7 Hz, 2H, H3’ and H5’), 4.79 (s, 2H, O*CH_2_* CH_2_N), 3.98 (t, *J*=6.5 Hz, 2H, O*CH_2_* CH_2_CH_3_), 3.90 (s, 3H, OCH_3_), 3.07 (s, 2H, OCH_2_ *CH_2_* N), 2.58 (s, 4H, N*CH_2_* CH_3_×2), 1.85–1.78 (m, 2H, OCH_2_ *CH_2_* CH_3_), 1.22–1.14 (m, 6H, NCH_2_ *CH_3_* ×2), 1.04 ppm (t, *J*=7.4 Hz, 3H, OCH_2_CH_2_ *CH_3_*).^13^C NMR (101 MHz, CDCl_3_): δ_C_ 165.46, 160.93, 157.89, 157.38, 147.61, 130.57, 129.57, 129.16, 125.36, 115.31, 114.15, 101.43, 69.47, 55.60, 50.79, 47.94, 22.52, 11.76, 10.48 ppm. HPLC: 80 % CH_3_CN, 20 % H_2_O/0.1 Et_2_NH, rt=9.78 min.

**6‐Methoxy‐4‐(2‐piperidin‐1‐ylethoxy)‐2‐(4‐propoxyphenyl)quinazoline (12 b)**. General procedure D (time=3 h): starting from derivative **26** (0.30 g, 0.79 mmol) and using K_2_CO_3_ (0.40 g, 2.91 mmol) and 1‐(2‐chloroethyl)piperidine hydrochloride (0.36 g, 1.94 mmol), compound **12 b** was obtained, after extraction with EtOAc and purification by flash chromatography column (EtOAc/MeOH 98/2), as a white solid in 25 % yield (0.10 g); mp=86.0–86.5 °C . ^1^H NMR (400 MHz, CDCl_3_): δ_H_ 8.45 (d, *J*=8.7 Hz, 2H, H2’ and H6’), 7.83 (d, *J*=9.0 Hz, 1H, H8), 7.41–7.37 (m, 2H, H5 and H7), 6.97 (d, *J*=8.7 Hz, 2H, H3’ and H5’), 4.79 (t, *J*=6.2 Hz, 2H, O*CH_2_* CH_2_N), 3.98 (t, *J*=6.5 Hz, 2H, O*CH_2_* CH_2_CH_3_), 3.90 (s, 3H, OCH_3_), 3.07 (s, 2H, OCH_2_ *CH_2_* N), 2.81–2.70 (m, 4H, piperidine NCH_2_×2), 1.85–1.78 (m, 2H, OCH_2_ *CH_2_* CH_3_), 1.14–1.06 (m, 6H, piperidine CH_2_×2), 1.04 ppm (t, *J*=7.4 Hz, 3H, OCH_2_CH_2_ *CH_3_*).^13^C NMR (101 MHz, CDCl_3_): δ_C_ 165.54, 160.99, 157.98, 157.42, 147.67, 131.62, 129.64, 129.23, 125.30, 115.45, 114.22, 101.62, 69.54, 64.49, 57.48, 55.68, 55.09, 26.00, 24.14, 22.60, 10.55 ppm. HPLC: 80 % CH_3_CN, 20 % H_2_O/0.1 Et_2_NH, rt=10.59 min.

**4‐(2‐Azepan‐1‐ylethoxy)‐6‐methoxy‐2‐(4‐propoxyphenyl)quinazoline (12 c)**. General procedure D (time=3 h): starting from derivative **26** (0.30 g, 0.97 mmol) and using K_2_CO_3_ (0.40 g, 2.91 mmol) and 1‐(2‐chloroethyl)azepane hydrochloride (0.38 g, 1.94 mmol), compound **12 c** was obtained, after extraction with EtOAc and purification by flash chromatography (EtOAc/MeOH 99/1), as a white solid in 10 % yield (0.04 g); mp=95.0–96.5 °C. ^1^H NMR (400 MHz, CDCl_3_): δ_H_ 8.45 (d, *J*=8.9 Hz, 2H, H2’ and H6’), 7.83 (d, *J*=9.0 Hz, 1H, H8), 7.41–7.36 (m, 2H, H5 and H7), 6.97 (d, *J*=8.9 Hz, 2H, H3’ and H5’), 4.78 (t, *J*=6.2 Hz, 2H, O*CH_2_* CH_2_N), 3.98 (t, *J*=6.5 Hz, 2H, O*CH_2_* CH_2_CH_3_), 3.90 (s, 3H, OCH_3_), 3.10 (t, *J*=6.3 Hz, 2H, OCH_2_ *CH_2_* N), 2.86–2.84 (m, 4H, azepane NCH_2_×2), 1.87–1.78 (m, 2H, OCH_2_ *CH_2_* CH_3_), 1.67–1.58 (m, 7H, azepane CH_2_×4), 1.04 ppm (t, *J*=7.4 Hz, 3H, OCH_2_CH_2_ *CH_3_*).^13^C NMR (101 MHz, CDCl_3_): δ_C_ 165.58, 160.91, 157.93, 157.34, 147.59, 130.67, 129.57, 129.14, 125.23, 115.41, 114.15, 101.53, 69.47, 64.90, 55.90, 55.79, 55.58, 28.08, 26.99, 22.52, 10.47. HPLC: 80 % CH_3_CN, 20 % H_2_O/0.1 Et_2_NH, rt=16.67 min.

**(2‐{[2‐(5‐Chloro‐2‐thienyl)‐6‐methoxyquinazolin‐4‐yl]oxy}ethyl)diethylamine (13 a)**. General procedure D (time=4 h): starting from derivative **27** (0.66 g, 2.25 mmol) and using K_2_CO_3_ (0.93 g, 6.75 mmol) and (2‐chloroethyl)diethylamine hydrochloride (0.77 g, 4.50 mmol), compound **13 a** was obtained, after extraction with CH_2_Cl_2_ and purification by flash chromatography column (CH_2_Cl_2_/acetone 95/5), as a yellow solid in 33 % yield (0.04 g); mp=87.0–87.5 °C. ^1^H NMR (400 MHz, CDCl_3_): δ_H_ 7.82–7.79 (m, 2H, H4’ and H8), 7.44 (dd, *J*=2.9 and 9.1 Hz, 1H, H7), 7.39 (d, *J*=2.7 Hz, 1H, H5), 6.97 (d, *J*=3.9 Hz, 1H, H3’), 4.73 (t, *J*=6.3 Hz, 2H, O*CH_2_* CH_2_N), 3.94 (s, 3H, OCH_3_), 3.04 (t, *J*=6.3 Hz, 2H, OCH_2_ *CH_2_* N), 2.73 (q, *J*=7.1 Hz, 4H, N*CH_2_* CH_3_×2), 1.15 ppm (t, *J*=7.1 Hz, 6H, NCH_2_ *CH_3_* ×2).^13^C NMR (101 MHz, CDCl_3_): δ_C_ 165.72, 157.84, 154.04, 147.27, 142.75, 133.30, 129.10, 127.45, 127.24, 125.81, 115.91, 101.89, 65.60, 55.77, 51.04, 48.19, 12.29. HPLC: 80 % CH_3_CN, 20 % H_2_O/0.1 % Et_2_NH, rt=9.63 min.

**2‐(5‐Chloro‐2‐thienyl)‐6‐methoxy‐4‐(2‐piperidin‐1‐ylethoxy)quinazoline (13 b)**. General procedure D (time=4 h): starting from derivative **27** (0.10 g, 0.34 mmol) and using K_2_CO_3_ (0.14 g, 1.02 mmol) and 1‐(2‐chloroethyl)piperidine hydrochloride (0.13 g,0.68 mmol), compound **13 b** was obtained, after extraction with CH_2_Cl_2_ and purification by flash chromatography column (CH_2_Cl_2_/acetone 95/5), as a yellow solid in 75 % yield (0.50 g); mp=122.5–123.5 °C. ^1^H NMR (400 MHz, CDCl_3_): δ_H_ 7.83–7.79 (m, 2H, H4’ and H8), 7.45 (dd, *J*=2.9 and 9.1 Hz, 1H, H7), 7.39 (d, *J*=2.9 Hz, 1H, H5), 6.97 (d, *J*=3.9 Hz, 1H, H3’), 4.80 (t, *J*=6.2 Hz, 2H, O*CH_2_* CH_2_N), 3.95 (s, 3H, OCH_3_), 2.94 (t, *J*=6.2 Hz, 2H, OCH_2_ *CH_2_* N), 2.65–2.58 (m, 4H, piperidine N*CH_2_* ×2), 1.66–1.62 (m, 4H, piperidine CH_2_×2), 1.51–1.48 ppm (m, 2H, piperidine CH_2_).^13^C NMR (101 MHz, CDCl_3_): δ_C_: 165.65, 157.95, 154.14, 147.27, 142.71, 133.32, 129.10, 127.46, 127.27, 125.74, 115.92, 101.99, 64.91, 57.48, 55.81, 55.17, 26.11, 24.26. HPLC: 80 % CH_3_CN, 20 % H_2_O/0.1 Et_2_NH, rt=10.34 min.

**2‐Bromobenzamide (30)**.^[42]^ 2‐Bromobenzoic acid **29** (2.20 g, 10.90 mmol) was added to SOCl_2_ (2.00 mL, 27.56 mmol) and the mixture was stirred at reflux for 90 min. After removing of the excess of SOCl_2_ under vacuum, a brown oil was obtained and immediately added at 0 °C to a solution of 33 % aq. NH_3_ (2.20 mL) in CH_3_CN (60 mL). The reaction mixture was stirred at room temperature for 1 h and then concentrated under vacuum. The resulting mixture was poured into ice/water and the pH was modified up to 5 by 2 N HCl to give a precipitate that was filtrated. Target compound **30** ^[42]^ was obtained as a white solid in 95 % yield (2.07 g). ^1^H NMR (400 MHz, Acetone): δ_H_ 7.60 (dd, *J*=1.0 and 7.8 Hz, 1H, H6), 7.45 (dd, *J*=1.7 and 7.6 Hz,1H, H3), 7.38 (dt, *J*=1.2 and 7.5 Hz, 1H, H5), 7.30 (dt, *J*=1.7 and 7.4 Hz, 1H, H4), 7.20–7.20 (m, 1H, NH_2_), 6.89–6.83 ppm (m, 1H, NH_2_).

**3‐(4‐Propoxyphenyl)isoquinolin‐1‐ol (31)**. A pressure tube was charged with 2‐bromobenzamide **30** (0.50 g, 2.5 mmol), CuBr (0.04 g, 0.25 mmol), 1‐(4‐propoxyphenyl)ethan‐1‐one 4 (0.54 g, 3.00 mmol), and Cs_2_CO_3_ (1.63 g, 5.00 mmol) in dry DMSO (5 mL). The tube was saturated with Ar, closed, and stirred at 110 °C for 8 h. Then, the reaction mixture was cooled, poured into ice/water and acidified with 2 N HCl to pH=4. The aqueous mixture was extracted by EtOAc (x3) and the organic layer was washed H_2_O (x2), brine (x2), and dried over Na_2_SO_4_. After evaporation, the residue was triturated with Et_2_O to give a yellow precipitate which was filtered to afford the title compound **31** as a yellow solid in 30 % yield (0.20 g). ^1^H NMR (400 MHz, DMSO‐*d_6_*): δ_H_ 11.40 (brs, 1H, OH), 8.14 (d, *J*=7.6 Hz, 1H, H8), 7.75–7.55 (m, 4H, H5, H6, H2’ and H6’), 7.41 (t, *J*=7.6 Hz, 1H, H7), 6.98 (d, *J*=8.2 Hz, 2H, H3’ and H5’), 6.80 (s, 1H, H4), 3.95 (t, *J*=6.3 Hz, 2H, O*CH_2_* CH_2_CH_3_), 1.75–1.60 (m, 2H, OCH_2_ *CH_2_* CH_3_), 0.95 ppm (t, *J*=7.2 Hz, 3H, OCH_2_CH_2_ *CH_3_*).

***N,N*** **‐diethyl‐2‐[(3‐(4‐propoxyphenyl)isoquinolin‐1‐yl)oxy]ethanamine (9 a)**. A mixture of 3‐(4‐propoxyphenyl)isoquinolin‐1‐ol **31** (0.30 g, 1.07 mmol) in POCl_3_ (2.00 mL, 21.4 mmol) was stirred under reflux for 3 h. POCl_3_ was distilled and the residue was cooled at 0 °C by an ice bath and triturated with saturated NaHCO_3_ solution to form a brown precipitate that was filtered to afford 1‐chloro‐3‐(4‐propoxyphenyl)isoquinoline **32**, which was immediately reacted in the next reaction step. Under N_2_ atmosphere, to a mixture of NaH (0.18 g, 4.50 mmol) in dry THF (10 mL), 2‐(diethylamino)ethan‐1‐ol (0.53 mL, 4.00 mmol) was added and the reaction mixture was stirred at room temperature until the end of bubbling. Then, intermediate **32** (0.30 g, 1.00 mmol) dissolved in dry THF (5 mL) was added dropwise at room temperature and the resulting mixture was stirred at reflux. After 3 h, the reaction mixture was cooled to 0 °C and quenched with EtOAc and water. Then, the resulting mixture was extracted by EtOAc (x3) and the organic layer was washed with brine, dried over Na_2_SO_4_ and evaporated to dryness to give a brown oil. After purification by flash column chromatography (Buchi Reveleris‐X2) (CH_2_Cl_2_/MeOH – gradient 1 to 8 % over 20 min), compound **9 a** was obtained as a yellow oil in 53 % yield (0.20 g); mp=63.5–64.5 °C. ^1^H NMR (400 MHz, CDCl_3_): δ_H_ 8.18 (d, *J*=8.2 Hz, 1H, H8), 8.08 (d, *J*=8.8 Hz, 2H, H2’ and H6’), 7.72 (d, *J*=8.1 Hz, 1H, H5), 7.65–7.55 (m, 3H, H4 and H6), 7.45 (dt, *J*=0.8 and 8.1 Hz, 1H, H7), 6.97 (d, *J*=8.8 Hz, 2H, H3’ and H5’), 4.75 (t, *J*=6.2 Hz, 2H, O*CH_2_* CH_2_N), 3.95 (t, *J*=6.3 Hz, 2H, O*CH_2_* CH_2_CH_3_), 3.10 (t, *J*=6.2 Hz, 2H, OCH_2_ *CH_2_* N), 2.75 (q, *J*=7.0 Hz, 4H, N*CH_2_* CH_3_×2), 1.90–1.75 (m, 2H, OCH_2_ *CH_2_* CH_3_), 1.15 (t, *J*=7.0 Hz, 6H, NCH_2_ *CH_3_* ×2), 1.05 ppm (t, *J*=7.4 Hz, 3H, OCH_2_CH_2_ *CH_3_*). ^13^C NMR (100 MHz, CDCl_3_): δ_C_ 159.61, 159.50, 147.56, 138.91, 131.71, 130.38, 127.73, 126.34, 125.85, 124.02, 118.38, 114.44, 109.07, 69.50, 63.97, 51.02, 47.84, 22.54, 11.75, 10.49 ppm. HPLC: 80 % CH_3_CN, 20 % H_2_O/0.1 Et_2_NH, rt=10.83 min.

**1‐(2‐(Piperidin‐1‐ylethoxy)‐3‐(4‐propoxyphenyl)isoquinoline (9 b)**. Following the procedure used to prepare compound **9 a** and using 1‐piperidinethanol (reaction time: 1 h), after purification by flash column chromatography (Buchi Reveleris‐X2) (CH_2_Cl_2_/MeOH – gradient 1 to 8 % over 20 min), the title compound **9 b** was obtained as a low‐melting white solid in 50 % yield (0.20 g); mp=80.5–81.5 °C. ^1^H NMR (400 MHz, CDCl_3_): δ_H_ 8.20 (d, *J*=8.2 Hz, 1H, H8), 8.07 (d, *J*=7.4 Hz, 2H, H2’ and H6’), 7.72 (d, *J*=8.1 Hz, 1H, H5), 7.65–7.55 (m, 2H, H4 and H6), 7.46 (t, *J*=8.1 Hz, 1H, H7), 6.97 (d, *J*=7.6 Hz, 2H, H3’ and H5’), 4.79 (t, *J*=5.6 Hz, 2H, O*CH_2_* CH_2_N), 3.97 (t, *J*=6.8 Hz, 2H, O*CH_2_* CH_2_CH_3_), 2.95 (t, *J*=5.6 Hz, 2H, OCH_2_ *CH_2_* N), 2.75–2.55 (m, 4H, piperidine NCH_2_×2), 1.90–1.75 (m, 2H, OCH_2_ *CH_2_* CH_3_), 1.70–1.55 (m, 4H, piperidine CH_2_×2), 1.50–1.40 (m, 2H, piperidine CH_2_), 1.05 ppm (t, *J*=7.4 Hz, 3H, OCH_2_CH_2_ *CH_3_*).^13^C NMR (100 MHz, CDCl_3_): δ_C_ 159.63, 159.49, 147.58, 138.89, 131.74, 130.35, 127.73, 126.32, 125.82, 124.07, 118.42, 114.44, 108.96, 69.50, 63.87, 57.72, 54.94, 25.92, 24.10, 22.54, 10.49 ppm. HPLC: 80 % CH_3_CN, 20 % H_2_O/0.1 Et_2_NH, rt=12.25 min.

**2‐Chloro‐4‐(4‐propoxyphenyl)quinazoline (34)**. In a MW vial, to mixture of 2,4‐dichloroquinazoline **33** (0.55 g, 2.75 mmol), 4‐propoxyphenylboronic acid (0.50 g, 2.75 mmol), Pd(PPh_3_)_4_ (0.16 g, 0.10 mmol) in DME (10 mL), aq. 2 M sol. of Na_2_CO_3_ (2.75 mL, 5.50 mmol) was added. Then, reaction mixture was degassed purging with Ar and irradiated by MW at the following conditions: time 15 min, max pressure 8 bar, cooling ON, temperature 100 °C. Then, reaction mixture was washed with brine, diluted with CHCl_3_, dried over Na_2_SO_4_ and evaporated to dryness to give crude solid. After trituration with MeOH, compound **34** was obtained as a white solid in 72 % yield (0.60 g). ^1^H NMR (400 MHz, CDCl_3_): δ_H_ 8.16 (d, *J*=8.4 Hz, 1H, H5), 7.98 (d, *J*=8.8 Hz, 1H, H8), 7.87 (t, *J*=8.8 Hz, 1H, H7), 7.80–7.73 (m, 2H, H2′ and H6′), 7.58 (dt, *J*=0.9 and 8.4 Hz,1H, H6), 7.10–7.04 (m, 2H, H3′ and H5′), 4.00 (t, *J*=6.7 Hz, 2H, O*CH_2_* CH_2_CH_3_), 1.95–1.80 (m,2H, OCH_2_ *CH_2_* CH_3_),1.05 ppm (t, *J*=7.1 Hz, 3H, OCH_2_CH_2_ *CH_3_*). ^1^H NMR 2D NOESY (400 MHz, CDCl_3_) showed a relevant cross‐peak indicating H5→H2′/H6′ correlation.

***N***, ***N*** **‐diethyl‐2‐((4‐(4‐propoxyphenyl)quinazolin‐2‐yl)oxy)ethanamine (10 a)**. Under N_2_ atmosphere, to a mixture of 60 % NaH (0.05 g, 1.34 mmol) in dry THF (5 mL), 2‐(diethylamino)ethan‐1‐ol (0.18 mL, 1.34 mmol) was added. After the end of bubbling, a solution of intermediate **34** (0.20 g, 0.67 mmol) in dry THF (5 mL) was added dropwise and the reaction mixture was stirred at reflux for 1 h. Then, it was cooled at 0 °C, quenched with EtOAc and water, and then concentrated under vacuum. The aqueous mixture was extracted with EtOAc (x3) and the organic layer was washed with water, brine, dried over Na_2_SO_4_, and evaporated to dryness to give a crude yellow oil. After purification by flash column chromatography (Buchi Reveleris‐X2) (CH_2_Cl_2_/MeOH – gradient 1 to 7 % over 20 min) compound **10 a** was obtained as a light yellow solid in 55 % yield (0.10 g); mp=91.5–93.0 °C. ^1^H NMR (400 MHz, CDCl_3_): δ_H_ 8.05 (dd, *J*=0.7 and 8.2 Hz, 1H, H5), 7.83 (d, *J*=8.0 Hz, 1H, H8), 7.78–7.72 (m, 3H, H7, H2′ and H6′), 7.36 (dt, *J*=1.3 and 8.2 Hz, 1H, H6), 7.05–7.00 (m, 2H, H3′ and H5′), 4.63 (t, *J*=6.8 Hz, 2H, O*CH_2_* CH_2_N), 4.00 (t, *J*=6.5 Hz, 2H, O*CH_2_* CH_2_CH_3_), 3.00 (t, *J*=6.8 Hz, 2H, OCH_2_ *CH_2_* N), 2.68 (q, *J*=7.1 Hz, 4H, N*CH_2_* CH_3_×2), 1.90–1.78 (m, 2H, OCH_2_ *CH_2_* CH_3_), 1.15–1.00 ppm (m, 9H, OCH_2_CH_2_ *CH_3_* and NCH_2_ *CH_3_* ×2).^13^C NMR (101 MHz, CDCl_3_): δ_C_ 171.29, 161.74, 160.91, 153.21, 133.79, 131.74, 129.15, 127.59, 127.21, 124.56, 120.01, 114.39, 69.66, 65.19, 51.11, 47.72, 22.53, 11.83, 10.54 ppm. HPLC: 80 % CH_3_CN, 20 % H_2_O/0.1 Et_2_NH, rt=5.60 min.

**2‐(2‐(piperidin‐1‐ylethoxy)‐4‐(4‐propoxyphenyl)quinazoline (10 b)**. Following the procedure used to prepare compound **10 a** and using 1‐piperidinethanol (reaction time 1 h), after purification by flash chromatography column (Buchi reveleris‐X2) (CH_2_Cl_2_/MeOH – gradient 1 to 7 % over 20 min), the title compound **10 b** was obtained as a low‐melting white solid in 50 % yield (0.10 g); mp=82.5–83.5 °C. ^1^H NMR (400 MHz, CDCl_3_): δ_H_ 8.05 (dd, *J*=1.8 Hz, 1H, H5), 7.82 (d, *J*=8.0 Hz, 1H, H8), 7.78–7.70 (m, 3H, H7, H2′ and H6′), 7.35 (dt, *J*=1.3 and 8.2 Hz, 1H, H6), 7.05–7.00 (m, 2H, H3′ and H5′), 4.67 (t, *J*=6.2 Hz, 2H, O*CH_2_* CH_2_N), 4.00 (t, *J*=6.5 Hz, 2H, O*CH_2_* CH_2_CH_3_), 2.92 (t, *J*=6.8 Hz, 2H, OCH_2_ *CH_2_* N), 2.65–2.50 (m, 4H, piperidine NCH_2_×2), 1.91–1.79 (m, 2H, OCH_2_ *CH_2_* CH_3_), 1.65–1.55 (m, 4H, piperidine CH_2_×2), 1.05 ppm (t, *J*=6.9 Hz, 3H, OCH_2_CH_2_ *CH_3_*). ^3^C NMR (101 MHz, CDCl_3_): δ_C_ 171.29, 161.70, 160.91, 153.19, 133.79, 131.74, 129.10, 127.58, 127.22, 124.57, 120.02, 114.40, 69.66, 64.90, 57.49, 54.88, 25.78, 24.10, 22.53, 10.54. HPLC: 80 % CH_3_CN, 20 % H_2_O/0.1 Et_2_NH, rt=5.44 min.

## Conflict of interest

The authors declare no conflict of interest.
